# Drought stress resistance indicators of chickpea varieties grown under deficit irrigation conditions

**DOI:** 10.7717/peerj.14818

**Published:** 2023-03-10

**Authors:** Ali Beyhan Ucak, Hüseyin Arslan

**Affiliations:** 1 Siirt University, Faculty of Agriculture, Department of Biosystems Engineering, Siirt, Türkiye; 2 Harran University, Faculty of Agriculture Department of Field Crops, Şanlıurfa, Türkiye

**Keywords:** Chickpea, Drought, Resistant, Plant water stress index, Chlorophyll content

## Abstract

The aim of this study was to determine the drought stress resistance of three chickpea cultivars (Inci, Hasanbey and Seçkin) grown under water deficit conditions and to discuss the use of yield, crop water stress index and chlorophyll index values as drought stress tolerance indicators in breeding studies. Three drought stress levels, (full irrigation = no stress - I_100_, deficit irrigation = moderate stress - I_50_, and no irrigation = severe stress - I_0_) were used as irrigation treatments. The highest seed yield (1,984 kg ha^−1^) in severe stress conditions was recorded for the Inci cultivar with a low crop water stress index (CWSI) (0.50) and high chlorophyll index (33.60 SPAD). The lowest seed yield (1,783.66 kg ha^−1^) in I_0_treatment was noted for the Seçkin cultivar which had a high CWSI (0.58) and low chlorophyll index (32.88 SPAD). The highest seed yield (2,566.33 kg ha^−1^) in full irrigation was recorded for the Inci cultivar which had a low CWSI (0.19) and high chlorophyll index (44.39 SPAD), while the lowest seed yield (2,328.00 kg ha^−1^) in I_100_ treatment was recorded for the Seçkin cultivar which had a high CWSI (0.26) and low chlorophyll index (42.12 SPAD). The seed yield of the Hasanbey cultivar in both severe stress (1,893 kg ha^−1^) and full irrigation (2,424.00 kg ha^−1^) conditions was between Inci and Seçkin varieties. The chlorophyll index and yield had a significant positive (*r* = 0.877) correlation, while a significant negative (*r* = −0.90) relationship was determined between CWSI and yield. Seed yield of the Inci cultivar in I_0_and I_100_treatments and water use efficiency revealed that the Inci cultivar is resistant to drought stress. Therefore, the Inci cultivar can be used in drought stress tolerance studies. In addition, the CWSI and chlorophyll index values can be employed as resistance indicators in chickpea breeding studies to determine the drought resistant chickpea cultivars.

## Introduction

Water use in agriculture is in a constant competition with industrial uses. Increasing demand for water use decreases groundwater resources, pollutes water ecosystems and deteriorates the quality. Therefore, improvement of new water resources becomes highly expensive (Aküzüm, Çakmak & Gökalp, 2003). Limited water and soil resources under current conditions necessitates higher water use efficiency (Cakmak & Gökalp, 2013). Drought stress is a meteorological phenomenon, caused by extended period without precipitation (Kalefetoglu & Ekmekci, 2005). The severity of drought stress depends on water retention capacity of soils and evapotranspiration rate of plants. The decrease in quantity and quality of water resources caused severe drought stress. Therefore, cultivation of drought resistant crop species or cultivars is needed to alleviate water shortage or severe drought events. Studies on identifying the tolerance mechanisms of plant species to stress conditions, and protection and transfer of plant gene resources are highly important (Ors & Ekinci, 2015; Kalefetoglu & Ekmekci, 2005). Considering the global climate change and associated negative effects on agriculture, determination and development of new chickpea genotypes that can be grown under drought stress conditions is important to sustain production. Determining new chickpea genotypes resistant to drought stresses or development of new varieties using the identified resistant/tolerant materials will provide valuable foundation for future breeding studies.

Drought stress suppresses plant growth due to limited moisture availability. The first symptoms of drought stress appear at stomata level, where stomatal closure prevents water losses by transpiration (Elsheery & Cao, 2008; Helaly et al., 2017; Helaly et al., 2022; Omar et al., 2018; Flexas & Medrano, 2002). Stomatal closure also reduces CO_2_ availability at chloroplast level; thus, adversely impacts net photosynthesis (Cornic, 2000). Internal water status of plants can be determined accurately using crop water stress index (CWSI) compared to water content of soils and atmospheric demand (Reginato & Howe, 1985; Gençoğlan & Yazar, 1999; Irmak, Haman & Bastug, 2000). The changes in leaf temperature depend on the transpiration of plants’ air temperature (Ta). The increase in the transpiration rate significantly decreases leaf temperature, which could be lower than the Ta (Elsheery & Cao, 2008; Helaly et al., 2017; Helaly et al., 2022; Omar et al., 2018; Elsheery et al., 2020a; Elsheery et al., 2020b; Naser et al., 2016; Mohamed et al., 2021). The difference in leaf temperature and Ta and psychrometric measurements are used to determine CWSI (Gencel, 2009; Irmak, Haman & Bastug, 2000; Orta, Erdem & Erdem, 2002). The CWSI values change with atmospheric temperature and soil water content (Reginato, 1983; Zia et al., 2012). Low canopy temperature (−5 to −1 °C) would be sufficient for plant growth. However, the Ta equal to or higher than the canopy temperature (Tc) induces water stress (Gençoğlan & Yazar, 1999; Gencel, 2009; Orta, Erdem & Erdem, 2002). Leaf air temperature (Tc-Ta) difference is an important indicator of plant drought stress (Jackson, 1982; Elsheery et al., 2007; Elsheery, Wilske & Kun-Fang, 2008; Elsheery et al., 2020c; Yan et al., 2021). Choudhury & Idso (1984), Chen, Lin & Lü (2010), Kar & Kumar (2010), and Zia et al. (2013) reported that air and dew temperatures are effective on canopy temperature under high soil water content.

Studies on drought tolerance are extremely important for plant breeders. Thus, leaf temperature and duration of the green period for the shoots are the most important characteristics to measure drought tolerance under low soil water availability. Canopy temperature is the most reliable method to measure drought stress and can be employed as criteria in plant breeding studies (Moroni et al., 2012). Generally, decrease in soil moisture before irrigation increases plant leaf temperature which increases CWSI values (Kirnak & Gencoglan, 2001). Previous studies reported that lines or varieties with low CWSI and high chlorophyll index are high-yielding, while those with low CWSI and high chlorophyll index are low-yielding (Ucak et al., 2016). Bahar et al. (2008) found a significant correlation between leaf temperature (canopy temperature) values and yield of bread and durum wheat varieties grown in Cukurova region, Turkey, and concluded that these parameter could be used as a selection criterion in wheat breeding. The parameters used to determine the drought or water stress should be reproducible and obtained easily, fast and incur less cost. Chlorophyll index is one of the most important characteristics in determining drought tolerance. Camoglu, Genç & Aşık (2011) reported that leaf water and chlorophyll content can be used for the instant drought stress determination. The findings of Ucak et al. (2016) who investigated drought tolerance in sweet corn lines revealed that soil moisture and chlorophyll index had a significant positive correlation.

Drought-resistant varieties have been determined in wheat (Sharma & Duveiller, 2006; Zia et al., 2012), rice (Weerakoon, Maruyama & Ohba, 2008), cotton (Cottee et al., 2010), and sunflower (Ucak et al., 2014), whereas the response of chickpea plants to drought stress has been rarely studied (Wang et al., 2006; Cancı& Toker, 2009; Ceyhan et al., 2012). In addition, the lack of effective selection criteria in agronomic studies to differentiate the potential status of chickpea varieties under drought stress is one of the most important limiting factors in agronomic or breeding studies. The aim of this research was to measure the leaf crown temperature values of chickpea genotypes at different irrigation levels (in open field conditions, not greenhouse) and to determine the CWSI values by using these measured leaf crown temperature values and to determine their drought stress tolerance according to the determined CWSI values and to examine the use of CWSI values as a stress tolerance indicator in drought studies. The CWSI value can vary between 0 and 1. The CWSI values approaching 0 are considered drought-resistant, while those approaching 1 will be considered drought-sensitive (Ucak et al., 2014).

## Materials and Methods

The field experiment was conducted during chickpea growing season of 2015 in Siirt Province, Turkey. The altitude of the experimental site is 894 m and is situated at 37°58′N latitude and 41°50′E longitude. The chickpea varieties used in the study were Inci, Hasanbey and Seçkin The genotypes were registered by Eastern Mediterranean Agricultural Research Institute, but they have not been tested for drought tolerance. Therefore, the genotypes were chosen to test the resistance to drought in arid and hot climatic conditions. The climate data for long term and during growing season are presented in Table 1. The study area has a typical continental climate characterized by cold and rainy winters, and dry and hot summer seasons.

**Table 1:** Climate data of the experimental site.

Climate parameter	Climate data long-term (2005–2014)							
	February	March	April	May	June	July	August	September
Mean maximum temperature (°C)	13.5	21.3	26.4	27.8	33.0	37.2	36.0	32.7
Mean temperature (°C)	5.3	10.2	14.3	20.2	27.1	31.1	31.0	25.4
Mean minimum temperature (°C)	−3.4	2.6	5.1	9.0	17.8	23.4	27.0	14.7
Mean relative humidity (%)	68.0	57.6	59.8	49.3	34.9	30.3	29.5	37.4
Mean wind speed (m s^−1^*)*	0.8	1.3	1.1	1.0	1.1	1.1	1.0	1.0
Monthly total sunshine (h)	104.6	160.2	201.1	277.2	309.8	363.0	330.6	244.3
Total precipitation (mm)	85.5	78.9	118.2	36.9	11.5	0.6	2.7	7.9
	**Climate data for during growing season (2015)**							
Mean maximum temperature (°C)	10.7	14.4	19.0	26.4	33.1	39.1	38.4	35.2
Mean temperature (°C)	6.0	9.1	13.7	20.4	26.8	32.0	31.4	28.2
Mean minimum temperature (°C)	2.4	5.2	9.1	14.5	20.0	24.6	24.2	21.5
Mean relative humidity (%)	70.8	63.1	55.8	43.0	27.8	19.6	22.5	22.9
Mean wind speed (m s^−1^*)*	0.7	1.2	1.1	1.0	1.2	1.0	1.1	1.0
Sunshine (h)	103.4	159.2	200.1	278.2	311.9	366.1	322.4	245.5
Precipitation (mm)	92.0	125.0	53.2	29.2	3.6	0.0	2.4	0.0

Three undisturbed and one disturbed soil samples were taken from three depths (0–30, 30–60 and 60–90 cm) before the experiment. Undisturbed soil samples were collected using a steel core sampler (100 cm^3^), were saturated and equilibrated to −1/3 bar matric potential for field capacity moisture content (Klute, 1986). Bulk density in undisturbed samples was determined using the core method (Blake & Hartge, 1986). The disturbed soil samples were used in organic matter content, texture and permanent wilting point moisture content analysis. Walkley-Black dichromate oxidation method was used to determine soil organic matter content (Tuzuner, 1990), and water content at a permanent wilting point (−15 bar) was determined according to Klute (1986). Soil texture (clay, silt and sand content) was determined using the hydrometer method (Tuzuner, 1990).

The soils of the experimental field had a clay texture (over 53% in 0–90 cm), with low electrical conductivity, slightly alkaline reaction and moderate organic matter content. The calcium carbonate content of the experiment was not high (6.4, 1.9 and 1.9% for 0–3 30–60 and 60–90 cm depth) to pose a problem for plant growth. Available phosphorus content was not sufficient for plant growth, while potassium content of soils was high. Mean field capacity, wilting point and available water content for 0–90 cm soil depth was 433, 312 mm and 121 mm, respectively. The average bulk density for 0–90 cm soil depth as 1.40 g cm^−3^.

Chemical properties of irrigation water (electrical conductivity (EC), pH anion and cation contents) were determined using the methods described in Tuzuner (1990). The EC of irrigation water 0.34 dS m^−1^ and pH was 7.21 (C_2_S_1_class) that indicate a high quality irrigation water. The irrigation water used in the experiment was not saline, alkaline or sodic to pose any damage for chickpea plants.

The layout of experiment was randomized complete blocks with split-plots. The chickpea varieties were placed in main plots, and irrigation levels were in sub-plots. Each plot had four rows with 30 cm interrow and 8–10 cm intrarow spacings. The size of plots were 1.2 m width and 6 m length (7.2 m^2^) with a 2 m buffer zone between the plots. Experimental field was prepared for sowing using two disc harrows, one ridge lister and a ridge roller. The harvested area in the middle two rows was used in the calculations of yield for each treatment. The amount of seeds thrown in a row is 60 pieces. Eighty percent of annual rainfall in the study area occurs in winter period, while the remaining falls in March and April. For this reason, this research was carried out as late winter planting in order to delay the precipitation period and to provide stress conditions.

The irrigation treatments used in the experiment were (i) full irrigation (I_100_) in which all water consumed was applied (I_100_, control), (ii) 50% irrigation (I_50_) where 50% of the consumed water was applied, and (iii) no irrigation (I_0_) where no water was applied. Irrigation was scheduled to be once every 7 days. Gravimetric method was used to determine moisture content within 90 cm of soil profile before each irrigation. In I_100_ treatment; irrigation water was applied to bring the moisture content in 90 cm depth to the field capacity moisture content.

Water was applied with drip irrigation system equipped with hard PE pipes (10 atm operation pressure and 63 mm outer diameter). Each line (70 cm) in a plot had a lateral. The infiltration rate of experimental soils was very slow (seven mm h^−1^) due to the heavy texture; therefore the drippers with 4 L h^−1^ flow rate with 1 atm operation pressure were used for irrigation. Drippers on each lateral were placed at 33 cm distances. The DAP (diammonium phosphate) fertilizer containing 18% nitrogen (N) and 46% phosphorus (P) is frequently used as base fertilizer (Erdin & Kulaz, 2014). Before planting, 150 kg/ha of DAP fertilizer was broadcasted on the plots and mixed with the soil using a harrow. Moisture content within 90 cm soil depth was determined gravimetrically prior to each irrigation. Water content at field capacity was used to determine the amount of irrigation water needed in each treatment. Field capacity was considered as full irrigation (I_100_) (control treatment). Therefore, soil samples from 0–30, 30–60 and 60–90 cm layers in each irrigation treatment were collected before the irrigation, and the dry weight (percent) of soil samples was determined. The moisture content in depth was calculated using the following Eq. (1).

(1) \begin{eqnarray*}d=(Pw-P{w}_{\mathrm{AW}})\times As\times D/100\end{eqnarray*}

In the equation; d is moisture content in soil depth (mm), Pw is field capacity moisture content (%), Pw_AW_ is moisture content (%) at permanent wilting point, As is bulk density of soil (g cm^−3^) and D is layer depth (mm).

Total amount of water for the effective root depth (dT) was calculated by adding up the calculated water content for each layer (Eq. (2)).

(2) \begin{eqnarray*}{d}_{\mathrm{T}}={d}_{\mathrm{(0- 30)}}+{d}_{\mathrm{(30- 60)}}+{d}_{\mathrm{(60- 90)}}\end{eqnarray*}

The volume of irrigation water for each plot was calculated by multiplying the size of a plot, the ratio of water deficit (1.0, 0.70, and 0.35) and the ratio of plant cover (Eq. (3)).

(3) \begin{eqnarray*}V={d}_{\mathrm{T}}\times A\times {U}_{\mathrm{o}}\times P\end{eqnarray*}

In the equation, V is the volume of water (L) to be applied, A is the size of a plot (m^2^), Uo is the ratio of water deficit (%) and P is the ratio of plant cover (%). The P was calculated by dividing the width of the plant canopy by the distance of row spacing. Plant cover ratio was considered constant (0.30) until plant cover reached 30%. Then, the P was calculated between 30 and 80% plant cover and again fixed when plant cover was over 80%. The amount of irrigation water used in plots was calculated based on the principles explained in Ucak et al. (2016). The amount of water used in the irrigation of experimental plots was measured throughout the experiment.

Plants were thinned when reached 15–20 cm, and middle-breaking were performed at the 8–9 leaves stage. Weed infestation was mild and controlled mechanically. Since the epidemic of the chickpea plant was not severe, the pesticide was not used.

The moisture content in the effective root zone was determined both at the beginning of the growing season and end of the harvest. Water use efficiency (WUE) of chickpea plants was calculated by the ratio of yield (kg ha^−1^) to seasonal evapotranspiration (mm) (Scott, 2000) as shown in Eq. (4).

(4) \begin{eqnarray*}WUE=Y/ETa\end{eqnarray*}

In the equation, WUE is the total water use efficiency (kg min mm^−1^), Y is yield (kg ha^−1^) for the irrigation treatments (for each chickpea variety), and Eta is the seasonal evapotranspiration (mm). Water consumption of plants was calculated using the water balance equation of Zeleke & Wade (2012) Eq. (5).

(5) \begin{eqnarray*}E{T}_{\mathrm{a}}=P+I-{R}_{\mathrm{f}}-{D}_{\mathrm{p}}\pm \Delta S\end{eqnarray*}

In the equation, ET_a_ is evapotranspiration (mm), P is precipitation (mm), I is the amount of irrigation water (mm), R_f_ is surface flow (mm), D_p_ is deep infiltration (mm), and ΔS is soil moisture variation (mm) in the root zone.

Since the drip flow rate opted for the study is lower than the infiltration rate of the soil, surface flow did not occur. The amount of irrigation water was only enough to bring the existing moisture to the field capacity, thus no infiltration occurred deeper than the root zone.

Plant water stress index (CWSI) and chlorophyll index (CI) of chickpea varieties were determined weekly during the growing season. The CWSI was calculated by the empirical method developed by Idso (1982) (Eq. (6));

(6) \begin{eqnarray*}CWSI=[(Tc-Ta)-LL]/UL-LL\end{eqnarray*}

In the equation, CWSI is the plant water stress index, Tc is canopy temperature (°C), Ta is air temperature (°C), LL is the lower limit of water stress where the transpiration is at potential rate, and UL is the upper limit of water stress where the plants do not transpire. The CWSI values vary between 0 and 1. The CWSI values close to 0 are considered drought-resistant, while those close to 1 are considered drought-sensitive (Ucak et al., 2014). In this study, the CWSI values up to 0.28 are considered as resistant, the CWSI values between 0.29 and 0.37 are moderately resistant, CWSI values 0.38 and 0.49 are moderately sensitive, and the CWSI values over 0.50 are sensitive. The CI was measured using a portable chlorophyll meter (Minolta SPAD-502, Osaka, Japan). The CI values were separately evaluated for each irrigation treatment (I0 and I100). The highest and lowest CI values were different for severe water stress (I0) and full irrigation (I100) treatment. For severe deficit irrigation treatment, the CI was considered high for a SPAD value between 33 and 34 and the CI was low for a SPAD value between 32 and 33. For full irrigation treatment, the CI was defined high for a SPAD value between 42 and 44 and the CI was low for a SPAD value between 40 and 42 (Ucak et al., 2014; Bahar et al., 2008; Yıldırım et al., 2009).

### Statistical analysis

The effect of irrigation levels on yield and water use efficiency of three chickpeas cultivars was assessed using variance analysis (ANOVA). The difference among the means where ANOVA denoted significant differences between the treatments was determined using the least significant difference test (LSD; *p* < 0.05). The relationship between the investigated traits was determined using a correlation test. All statistical analyses were carried out using JMP (version 5.0.1a) statistical software (Der & Everitt, 2002).

## Results

### Yield, chlorophyll index, and crop water stress index

**Figure 1:**
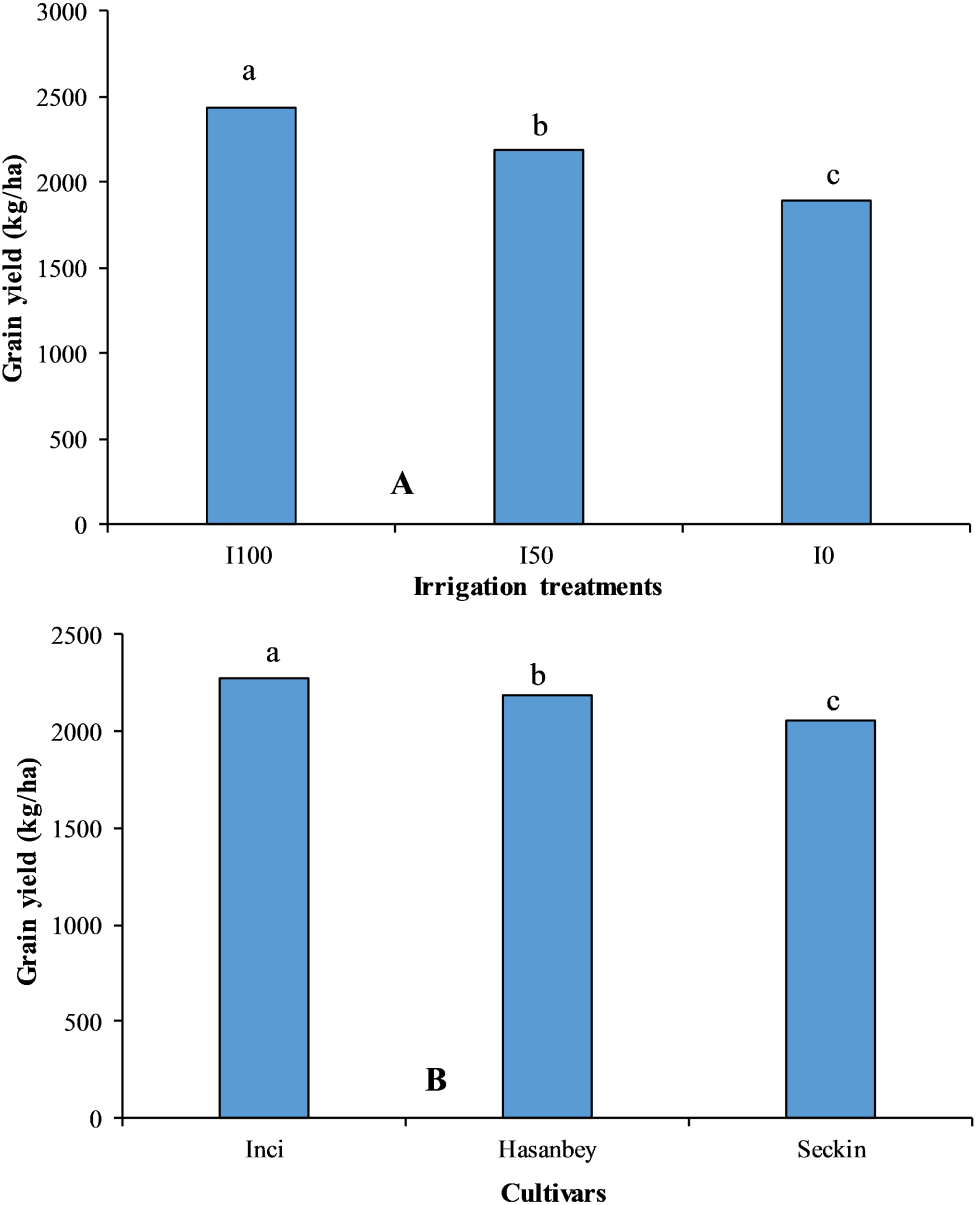
The impact of different irrigation treatments and cultivars on grain yield of chickpea. LSD values for irrigation treatments and cultivars are 15.57 and 18.56, respectively. Means sharing different letters are statistically different from each other ( *p* < 0.05).

**Figure 2:**
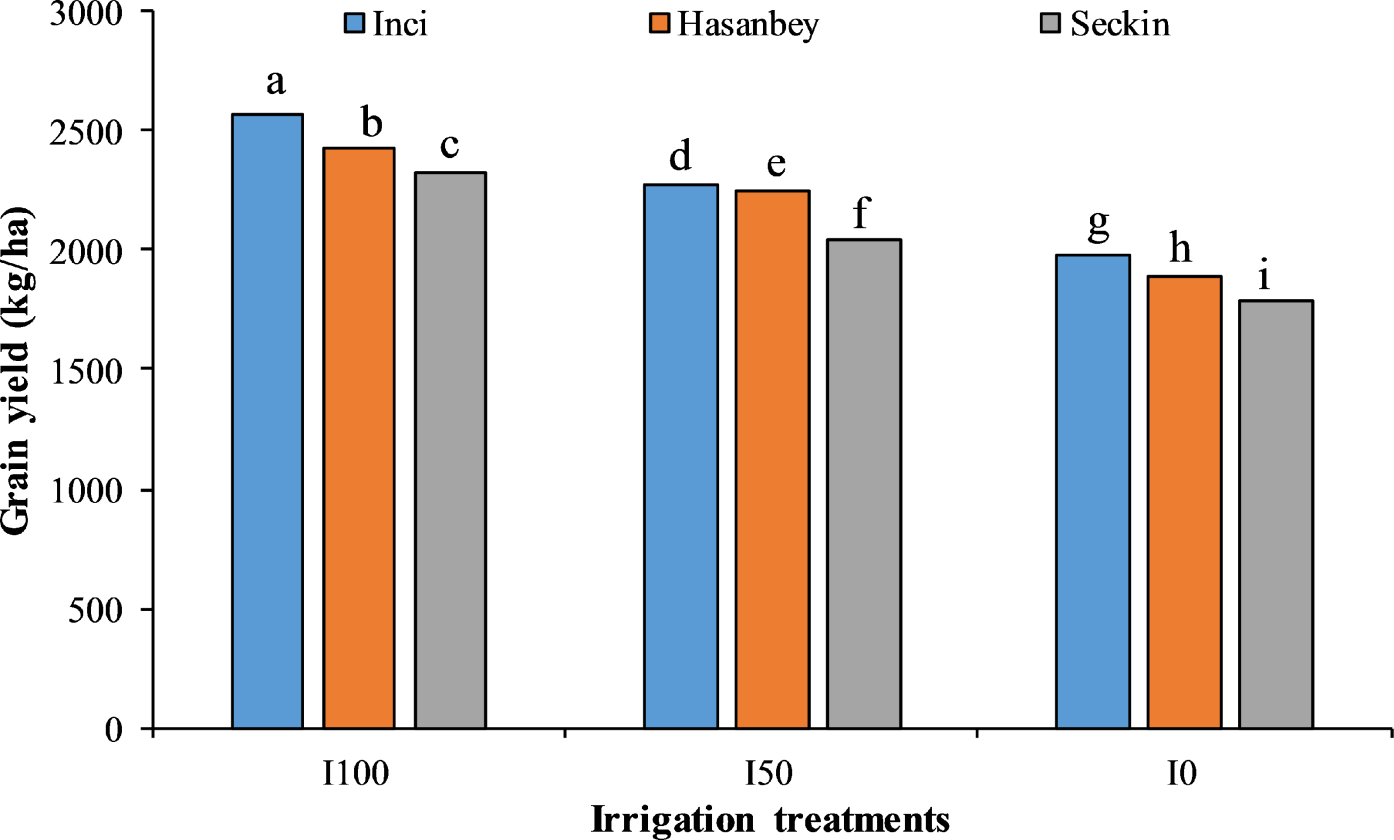
The interactive effect of different irrigation treatments and cultivars on grain yield of chickpea. LSD value for interaction is 26.97. Means sharing different letters are statistically different from each other ( *p* < 0.05).

The crop water stress index (CWSI), chlorophyll index (CI), plant root distribution (g), number of nodules (pieces), and water use efficiency (WUE) were significantly (*p* ≤ 0.01) affected by varieties, irrigation levels and the interactions of variety and irrigation levels (Table 2). The highest seed yield (1,984.00 kg ha^−1^) under extreme water stress (I_0_) treatment was noted for the Inci variety with low CWSI (0.50) and high CI (33.60 SPAD) values (Fig. 1). The lowest seed yield (1,784.00 kg ha^−1^) in I_0_ treatment was recorded for the Seçkin variety with high CWSI (0.58) and low CI (32.88 SPAD) values. The highest yield (2,566.00 kg ha^−1^) under no water stress (I_100_) treatment was obtained recorded for the Inci variety with the lowest CWSI (0.19) and the highest CI (44.39 SPAD). However, the lowest yield (2,328.00 kg ha^−1^) in no water stress treatment was noted for the Seçkin variety with high CWSI (0.26) and a low CI (42.12 SPAD) (Fig. 2).

**Table 2:** Mean values of chickpea varieties and other parameters investigated in the study.

Treatments	CWSI	Chlorophyll index (SPAD)	Irrigation water (mm)	ETa (mm)	WUE (kg da^−1^-mm)	Number of nodules (pieces)	Plant root distribution (g)
Irrigation treatments							
I_100_	0.23 c	43.34 a	103.00	418.00	0.57 c	10.21 a	7.62 a
I_50_	0.45 b	35.91 b	51.50	366.50	0.61 a	8.53 b	5.14 b
I_0_	0.54 a	33.11 c	0.00	315.00	0.59 b	6.94 c	4.21 c
CV (%)	2.66	0.72	0.00	0.00	2.56	2.04	2.39
LSD (0.05)	0.012	0.28	0.00	0.00	0.016	0.18	0.14
	**	**			**	**	**
Varieties							
Inci	0.37 c	38.12 a	0.00	0.00	0.62 a	9.26 a	5.99 a
Hasanbey	0.41 b	35.91 b	0.00	0.00	0.59 b	8.45 b	5.66 b
Seçkin	0.44 a	33.11 c	0.00	0.00	0.56 c	7.96 c	5.32 c
CV (%)	2.66	0.72	0.00	0.00	2.56	2.04	2.39
LSD (0.05)	0.010	0.52	0.00	0.00	0.010	0.14	0.17
	**				**	**	**
Varieties × irrigation treatments							
I_100_ × Inci	0.19 h	44.39 a	103	418	0.59 ns	11.15 a	8.26 a
I_100_ × Hasanbey	0.23 g	43.52 b	103	418	0.57 ns	9.82 b	7.75 b
I_100_ × Seçkin	0.26 f	42.12 c	103	418	0.54 ns	9.66 b	6.86 c
I_50_ × Inci	0.41 e	36.38 d	51.50	367	0.65 ns	9.13 c	5.26 d
I_50_ × Hasanbey	0.46 d	36.12 d	51.50	369	0.61 ns	8.66 d	5.12 d
I_50_ × Seçkin	0.48 c	35.24 e	51.50	367	0.58 ns	7.79 e	5.04 d
I_0_ × Inci	0.50 c	33.60 f	0.00	315	0.63 ns	7.51 e	4.44 e
I_0_ × Hasanbey	0.55 b	32.88 g	0.00	315	0.60 ns	6.88 f	4.12 f
I_0_ × Seçkin	0.58 a	32.88 g	0.00	315	0.56 ns	6.44 g	4.08 f
CV (%)	2.66	0.72	0.00	0.00	0.00	0.00	2.39
LSD (0.05)	0.019	0.48	0.00	0.00	ns	0.32	0.24
	**					**	**

**Notes.**

* *p* ≤ 0.05.

** *p* ≤ 0.01. It is important within the error limits. ns: not significant. Similar letters in the same column are not, significantly different from each other.

WUE Water use efficiencies CWSI Plant water stress index Eta Evapotranspiration

The highest CI (33.60 SPAD) was noted for the Inci variety under I_0_, while the lowest CI (32.88 SPAD) was observed for the Seçkin variety. The highest CI (44.39 SPAD) was recorded for the Inci genotype under I_100_ treatment, whereas the lowest CI (42.12 SPAD) was determined for the Seçkin variety.

The ANOVA indicated a significant effect of genotype on the CWSI. The highest CWSI (0.58) in I_0_ treatment was determined for the Seçkin variety, while the lowest (0.50) was noted for the Inci. The highest CWSI (0.26) in I_100_ treatment was determined for the Seçkin variety, while the lowest CWSI (0.19) was noted for the Inci variety. Overall, the highest (0.44) and the lowest (0.37) CWSI values were recorded for the Seçkin and Inci varieties, respectively.

The highest number of nodules (NON) (7.51) in I_0_ treatment was determined for the Inci variety, while the lowest (6.44) for the Seçkin. Similarly, the highest (11.15) and the lowest (9.66) NON in I_100_ treatment was noted for the Inci and Seçkin varieties, respectively. The ANOVA indicated a significant impact of variety on NON. Overall, the highest (9.26) and the lowest (7.96) NON were recorded for the Inci and Seçkin genotypes, respectively (Table 2). The NON showed an increasing trend as the amount of irrigation water applied increased.

The highest (4.44 g) and the lowest (4.08 g) plant root distribution (PRD) in I_0_ treatment was recorded for the Inci and Seçkin varieties, respectively. Similarly, the highest (8.26) and the lowest (6.86 g) PRD in I_100_ treatment was determined for the Inci and Seçkin genotypes, respectively. The ANOVA indicated a significant impact of variety on PRD and the highest (5.99 g) and the lowest (5.32 g) PRD was observed for the Inci and Seçkin varieties, respectively. The PRD values showed an increasing trend with increased amount of irrigation water applied.

### Water use efficiency (WUE)

The highest (0.63) and the lowest (0.56) WUE in I_0_ treatment was determined for Inci and Seçkin varieties, respectively. Similarly, Inci and Seçkin varieties recorded the highest (0.59) an and the lowest (0.54) WEU in I_100_ treatment. The ANOVA indicated a significant impact of variety on WEU. Overall, the Inci and Seçkin varieties noted the highest (0.62) and the lowest (0.56 kg da^−1^ mm^−1^) WEU (Table 2).

### Correlation analyses

The values of correlation coefficients (r) indicating the relationships between yield and CI, CWSI, ETa, NON, plant root distribution and WUE of are given in Table 3. Statistically significant (*p* ≤ 0.01) relationships were recorded among all traits. The CI and yield had a significant positive (*r* = 0.877**) correlation, while a significant negative (r = −0.90) relationship was determined between CWSI and yield. The results indicated a strong negative (r = −0.68**) correlation between WUE and yield. Similarly, a significant positive (*r* = 0.97**) relationship was found between CI and yield, while a significant negative (r = −0.94**) relationship was recorded between CWSI and yield. Plant water consumption (*r* = 0.779**), NON (*r* = 0.973**) and PRD (*r* = 0.887**) had significant (*p* ≤ 0.01) positive correlations with yield.

**Table 3:** The correlation coefficients of between chickpea yield and other parameters.

	Yield	CWSI	CC	ETa	WUE	NOD	PRD
Yield	1						
CWSI	−0.90^**^	1					
CC	0.877^**^	−0.988^**^	1				
ETa	0.779^**^	−0.962^**^	0.979^**^	1			
WUE	−0.684^*^	0.399	−0.472^*^	−0.601^**^	1		
NOD	0.973^**^	−0.912^**^	0.882^**^	0.799^**^	−0.082	1	
PRD	0.887^**^	−0.978^**^	0.987^**^	0.948^**^	−0.422^*^	0.894^**^	1

**Notes.**

* *p* < 0.05.

** *p* < 0.01.

ns Not significant WUE Water use efficiencies CWSI Plant water stress index CC Chlorophyll content NOD Number of nodules PRD Plant root distribution ETa Plant water consumption

## Discussion

The results revealed an important interaction between drought stress and tested chickpea varieties. The results are in accordance with the findings of Kassab, Ellil & El-Kheir (2012) who reported a significant interaction between watering regimes and type of cultivars. Crops are highly susceptible to water deficiency during flowering phases and substantial yield losses have been reported due to the water stress experienced during flowering period (Ali & Shui, 2009). Water stress, even for a short period may cause substantial losses in crop yields (Alahdadi, Oraki & Khajani, 2011). Drought stress during heading and pollination can adversely affect grain formation in chickpea (Hajhassani-Asl et al., 2009). Similarly, Bajehbaj (2010) reported that the relative water content of leaves, number of seeds per head, plant height and leaf area index of chickpea significantly decreased under water deficiency. Lack of sufficient moisture from budding to the end of the flowering period adversely affected the productivity of chickpea varieties (Pankovic et al., 1999). The results obtained in this study are in accordance with Hajhassani-Asl et al. (2009), Pankovic et al. (1999) and Chimenti, Pearson & Hall (2002). Darvishzadeh et al. (2010) emphasized that the most important selection index in determining drought tolerance of chickpea varieties is similar performance of varieties under drought stressed and stress-free. Therefore, the Inci variety exhibited similar performance under I _100_ andI_0_ treatments. Thus, the Inci variety could be considered as a drought resistant variety and used in the future breeding studies.

The variety had a significant impact on CI in the current study. The highest (38.12) and the lowest (33.11) CI was noted for the Inci and Seçkin varieties, respectively. The decrease in CI of drought-resistant varieties was lower under water deficit conditions compared to sensitive varieties. The resistance level of plants to stress may significantly differ, and even different varieties of the same crop may have varying resistance level (Win et al., 2011; Devasirvatham et al., 2012; Camoglu, Genç & Aşık, 2011). Chlorophyll and carotenoid content of plants have a significant relationship with intensity of light, nitrogen content, particle size distribution of soil, and crop varieties (Mini & Wahab, 2002).

The chlorophyll and proline contents of plants are commonly used criteria in determining the resistant level to abiotic stresses (Duran, Coşkun & Savaşkan, 2010). The decrease in leaf chlorophyll contents under drought stress was reported by several researchers (Demirtas & Kirnak, 2009; Fernandez, 1992; Fernandez, Perry & Flore, 1997). The results obtained for chlorophyll index values of chickpea varieties are in agreement with the earlier reports. The CWSI was low at beginning of vegetative period and the highest values were recorded during flowering period in no-stress treatment. The CWSI values showed a tendency to decrease after irrigation events. The differences in CWSI values can be attributed to the differences in irrigation methods and irrigation programs. In addition, as narrow or broad, hairy or hairless leaves of the plants, climate and soil characteristics, and cultivation techniques are other important factors affecting CWSI values (Alderfasi & Nielsen, 2001; Testi et al., 2008). Furthermore, the differences in CWSI can be owed to photosynthesis metabolism. The optimum water level in the cell is needed for optimum photosynthesis and functioning of photosystem reactions involving chlorophyll. Since there was no enough water in the soil profile (0–90 cm) of the excessive stress treatment, the amount of water transferred to the leaves through the transmission tissues was also low. Low water content in leaves causes an increase in leaf temperature. Consequently, the decrease in CWSI in I_100_ may be associated with the excess water content of plant leaves. Similarly, Kirnak & Gencoglan (2001) reported that the decrease in soil moisture content before irrigation increases leaf temperature. The findings are in accordance with the results reported by other researchers.

The findings of Ucak et al. (2016) and Ucak et al. (2017) who conducted studies in Siirt and Sakarya provinces on drought resistance of sweet corn lines are in agreement with our data. The lines with low CWSI and high CI values had higher sweet corn yield; thus, CWSI and CI were recommended for drought resistance selection criteria (Ucak et al., 2016; Ucak et al., 2017). Higher CWSI values and decreased CI values in drought stress treatments have been reported by Zlatev et al. (2010) who indicated that PSII photosynthetic electron transfer system is restrained under drought stress. Likewise, Khayatnezhad et al. (2011) reported decreased CI values in drought stress and reduction in maize yield. Chickpea yield and CI values had a positive correlation, while yield and CWSI had a negative correlation. Briefly, crop yield increased as CI increased and yield decreased as CWSI increased. However, the increase or decrease in CI and CWSI was not constant and changed depending water stress conditions and varieties. Therefore, the CWSI and CI values can be evaluated as stress screening parameters in the selection of drought resistant varieties. In previous studies, a linear relationship was reported between CI and yield and concluded that high yields can be obtained from varieties with high CI (Khayatnezhad & Gholamin, 2012; Ucak et al., 2017).

The WUE values showed a decreasing trend as the amount of irrigation water applied increased. The results reported by Mahmoud & Ahmed (2016) were in accordance with our findings. Mahmoud & Ahmed (2016) indicated that the WUE increased with decreased amount of irrigation water and WUE and yield of drought-resistant varieties were significantly higher compared to drought sensitive varieties. Similar results have been reported by Killi et al. (2017) who found that yields of C3 plants significantly decreased under water stress. The results obtained in this study are in accordance with those reported in earlier studies.

## Conclusions

The results revealed that drought stress-resistant varieties have low crop water stress index (CWSI) and high chlorophyll index (CI) values. Briefly, the yield and chlorophyll index (CI) of the Inci variety under stress-free and stress treatments were higher than those recorded for Hasanbey and Seçkin varieties. Therefore, the Inci variety can be considered resistant to drought conditions and can be used in resistance studies as the plant material. Consequently, the CWSI and CI values can be used as stress screening parameters (resistance indicator) in the selection of drought stress-resistant cultivars. Abiotic factors such as water stress resulting from global climate changes cause a significant decrease in the yields of cash crops. Therefore, the improvement or breeding of drought-stress resistant varieties is a permanent measure in the long run to prevent yield decreases under water stress. Selecting varieties or lines that are resistant to drought stress is a reliable means of success in breeding studies.

##  Supplemental Information

10.7717/peerj.14818/supp-1 Supplemental Information 1 Field and laboratory observations observations in the field include all the buds that need to be taken during the period from planting to harvest.

10.7717/peerj.14818/supp-2 Supplemental Information 2 Uninterpreted analysis of variance results The data obtained for each character was subjected to analysis of variance and tried to be interpreted.

10.7717/peerj.14818/supp-3 Supplemental Information 3 Relationship between the studied characters The positive or negative situations of the relationship between the examined characters were analyzed. It has been determined what kind of a relationship there is between the characters.

10.7717/peerj.14818/supp-4 Supplemental Information 4 15/5.000 Çeviri sonuçlarıstar_border analysis of variance The data obtained for each character was subjected to analysis of variance and tried to be interpreted.

10.7717/peerj.14818/supp-5 Supplemental Information 5 Variance analysis was performed in the Jump 5.01 statistical package program Since the experimental design was split plots in randomized blocks, firstly, raw data were entered in Excel in accordance with this experimental design. In other words, the raw data were arranged in such a way that analysis of variance was carried out. In the research, 3 irrigation subjects and 3 genotypes constitute the plant material. Therefore, the data were subjected to analysis of variance in the jump 5.01 computer package program in accordance with the experimental design of divided parcels in random blocks. After the analysis, the value of Probe >F (significance level) was checked in the Test wrt Random Effects table (Below).

10.7717/peerj.14818/supp-6 Supplemental Information 6 Meteorogical data

## Additional Information and Declarations

### Data Availability

The following information was supplied regarding data availability: The raw measurements are available in the Supplemental Files.
