# Supplementary material for: Drought stress resistance indicators of chickpea varieties grown under deficit irrigation conditions
Source: PeerJ. 2023 Mar 10;11:e14818. doi: 10.7717/peerj.14818 (PMC10010177; doi:10.7717/peerj.14818)
Supplement: Supplemental Information 2 — The data obtained for each character was subjected to analysis of variance and tried to be interpreted. [file peerj-11-14818-s002.rtf]

Response kök dað
Whole Model
Actual by Predicted Plot

Summary of Fit
 	 	
RSquare	0,996472	
RSquare Adj	0,992357	
Root Mean Square Error	0,132462	
Mean of Response	5,663333	
Observations (or Sum Wgts)	27	
Tests wrt Random Effects
Source	SS	MS Num	DF Num	F Ratio	Prob > F	
tek	0,07209	0,03604	2	2,0243	0,2470	
tek*ceþit&Random	0,07122	0,01781	4	1,0148	0,4381	
sul kon	56,011	28,0055	2	1596,091	<.0001	
ceþit	1,96682	0,98341	2	55,2306	0,0012	
ceþit*sul kon	1,35756	0,33939	4	19,3425	<.0001	
Residual by Predicted Plot


tek
Leverage Plot

Effect Test
Sum of Squares	F Ratio	DF	Prob > F	
0,07208889	2,0243	2	0,2470	
Denominator MS Synthesis: 
 tek*ceþit&Random
Least Squares Means Table
Level	Least Sq Mean	 	Std Error	Mean	
1	5,6077778		0,04447915	5,60778	
2	5,6500000		0,04447915	5,65000	
3	5,7322222		0,04447915	5,73222	

tek*ceþit&Random
Leverage Plot

Effect Test
Sum of Squares	F Ratio	DF	Prob > F	
0,07122222	1,0148	4	0,4381	
Denominator MS Synthesis: 
 Residual
Least Squares Means Table
Level	Least Sq Mean	 	Std Error	
1,Hasanbey	5,6466667		0,07647722	
1,Seçkin	5,2733333		0,07647722	
1,Ýnci	5,9033333		0,07647722	
2,Hasanbey	5,6633333		0,07647722	
2,Seçkin	5,3700000		0,07647722	
2,Ýnci	5,9166667		0,07647722	
3,Hasanbey	5,6833333		0,07647722	
3,Seçkin	5,3533333		0,07647722	
3,Ýnci	6,1600000		0,07647722	

sul kon
Leverage Plot

Effect Test
Sum of Squares	F Ratio	DF	Prob > F	
56,010956	1596,091	2	<.0001	
Denominator MS Synthesis: 
 Residual
Least Squares Means Table
Level	Least Sq Mean	 	Std Error	Mean	
I0	4,2177778		0,04415414	4,21778	
I100	7,6288889		0,04415414	7,62889	
I50	5,1433333		0,04415414	5,14333	
LSMeans Differences Student's t
Alpha=
0,050 t=
2,17881LSMean[i] By LSMean[j]
Mean[i]-Mean[j]
Std Err Dif
Lower CL Dif
Upper CL Dif	I0	I100	I50	
I0	0
0
0
0	-3,4111
0,06244
-3,5472
-3,2751	-0,9256
0,06244
-1,0616
-0,7895	
I100	3,41111
0,06244
3,27506
3,54716	0
0
0
0	2,48556
0,06244
2,3495
2,62161	
I50	0,92556
0,06244
0,7895
1,06161	-2,4856
0,06244
-2,6216
-2,3495	0
0
0
0	


Level				Least Sq Mean	
I100	A	 	 	7,6288889	
I50	 	B	 	5,1433333	
I0	 	 	C	4,2177778	

Levels not connected by same letter are significantly different

ceþit
Leverage Plot

Effect Test
Sum of Squares	F Ratio	DF	Prob > F	
1,9668222	55,2306	2	0,0012	
Denominator MS Synthesis: 
 tek*ceþit&Random
Least Squares Means Table
Level	Least Sq Mean	 	Std Error	Mean	
Hasanbey	5,6644444		0,04447915	5,66444	
Seçkin	5,3322222		0,04447915	5,33222	
Ýnci	5,9933333		0,04447915	5,99333	
LSMeans Differences Student's t
Alpha=
0,050 t=
2,77645LSMean[i] By LSMean[j]
Mean[i]-Mean[j]
Std Err Dif
Lower CL Dif
Upper CL Dif	Hasanbey	Seçkin	Ýnci	
Hasanbey	0
0
0
0	0,33222
0,0629
0,15758
0,50687	-0,3289
0,0629
-0,5035
-0,1542	
Seçkin	-0,3322
0,0629
-0,5069
-0,1576	0
0
0
0	-0,6611
0,0629
-0,8358
-0,4865	
Ýnci	0,32889
0,0629
0,15424
0,50354	0,66111
0,0629
0,48646
0,83576	0
0
0
0	


Level				Least Sq Mean	
Ýnci	A	 	 	5,9933333	
Hasanbey	 	B	 	5,6644444	
Seçkin	 	 	C	5,3322222	

Levels not connected by same letter are significantly different

ceþit*sul kon
Leverage Plot

Effect Test
Sum of Squares	F Ratio	DF	Prob > F	
1,3575556	19,3425	4	<.0001	
Denominator MS Synthesis: 
 Residual
Least Squares Means Table
Level	Least Sq Mean	 	Std Error	
Hasanbey,I0	4,1200000		0,07647722	
Hasanbey,I100	7,7533333		0,07647722	
Hasanbey,I50	5,1200000		0,07647722	
Seçkin,I0	4,0866667		0,07647722	
Seçkin,I100	6,8666667		0,07647722	
Seçkin,I50	5,0433333		0,07647722	
Ýnci,I0	4,4466667		0,07647722	
Ýnci,I100	8,2666667		0,07647722	
Ýnci,I50	5,2666667		0,07647722	
LSMeans Differences Student's t
Alpha=
0,050 t=
2,17881LSMean[i] By LSMean[j]
Mean[i]-Mean[j]
Std Err Dif
Lower CL Dif
Upper CL Dif	Hasanbey,I0	Hasanbey,I100	Hasanbey,I50	Seçkin,I0	Seçkin,I100	Seçkin,I50	Ýnci,I0	Ýnci,I100	Ýnci,I50	
Hasanbey,I0	0
0
0
0	-3,6333
0,10816
-3,869
-3,3977	-1
0,10816
-1,2356
-0,7644	0,03333
0,10816
-0,2023
0,26898	-2,7467
0,10816
-2,9823
-2,511	-0,9233
0,10816
-1,159
-0,6877	-0,3267
0,10816
-0,5623
-0,091	-4,1467
0,10816
-4,3823
-3,911	-1,1467
0,10816
-1,3823
-0,911	
Hasanbey,I100	3,63333
0,10816
3,39768
3,86898	0
0
0
0	2,63333
0,10816
2,39768
2,86898	3,66667
0,10816
3,43102
3,90232	0,88667
0,10816
0,65102
1,12232	2,71
0,10816
2,47435
2,94565	3,30667
0,10816
3,07102
3,54232	-0,5133
0,10816
-0,749
-0,2777	2,48667
0,10816
2,25102
2,72232	
Hasanbey,I50	1
0,10816
0,76435
1,23565	-2,6333
0,10816
-2,869
-2,3977	0
0
0
0	1,03333
0,10816
0,79768
1,26898	-1,7467
0,10816
-1,9823
-1,511	0,07667
0,10816
-0,159
0,31232	0,67333
0,10816
0,43768
0,90898	-3,1467
0,10816
-3,3823
-2,911	-0,1467
0,10816
-0,3823
0,08898	
Seçkin,I0	-0,0333
0,10816
-0,269
0,20232	-3,6667
0,10816
-3,9023
-3,431	-1,0333
0,10816
-1,269
-0,7977	0
0
0
0	-2,78
0,10816
-3,0156
-2,5444	-0,9567
0,10816
-1,1923
-0,721	-0,36
0,10816
-0,5956
-0,1244	-4,18
0,10816
-4,4156
-3,9444	-1,18
0,10816
-1,4156
-0,9444	
Seçkin,I100	2,74667
0,10816
2,51102
2,98232	-0,8867
0,10816
-1,1223
-0,651	1,74667
0,10816
1,51102
1,98232	2,78
0,10816
2,54435
3,01565	0
0
0
0	1,82333
0,10816
1,58768
2,05898	2,42
0,10816
2,18435
2,65565	-1,4
0,10816
-1,6356
-1,1644	1,6
0,10816
1,36435
1,83565	
Seçkin,I50	0,92333
0,10816
0,68768
1,15898	-2,71
0,10816
-2,9456
-2,4744	-0,0767
0,10816
-0,3123
0,15898	0,95667
0,10816
0,72102
1,19232	-1,8233
0,10816
-2,059
-1,5877	0
0
0
0	0,59667
0,10816
0,36102
0,83232	-3,2233
0,10816
-3,459
-2,9877	-0,2233
0,10816
-0,459
0,01232	
Ýnci,I0	0,32667
0,10816
0,09102
0,56232	-3,3067
0,10816
-3,5423
-3,071	-0,6733
0,10816
-0,909
-0,4377	0,36
0,10816
0,12435
0,59565	-2,42
0,10816
-2,6556
-2,1844	-0,5967
0,10816
-0,8323
-0,361	0
0
0
0	-3,82
0,10816
-4,0556
-3,5844	-0,82
0,10816
-1,0556
-0,5844	
Ýnci,I100	4,14667
0,10816
3,91102
4,38232	0,51333
0,10816
0,27768
0,74898	3,14667
0,10816
2,91102
3,38232	4,18
0,10816
3,94435
4,41565	1,4
0,10816
1,16435
1,63565	3,22333
0,10816
2,98768
3,45898	3,82
0,10816
3,58435
4,05565	0
0
0
0	3
0,10816
2,76435
3,23565	
Ýnci,I50	1,14667
0,10816
0,91102
1,38232	-2,4867
0,10816
-2,7223
-2,251	0,14667
0,10816
-0,089
0,38232	1,18
0,10816
0,94435
1,41565	-1,6
0,10816
-1,8356
-1,3644	0,22333
0,10816
-0,0123
0,45898	0,82
0,10816
0,58435
1,05565	-3
0,10816
-3,2356
-2,7644	0
0
0
0	


Level							Least Sq Mean	
Ýnci,I100	A	 	 	 	 	 	8,2666667	
Hasanbey,I100	 	B	 	 	 	 	7,7533333	
Seçkin,I100	 	 	C	 	 	 	6,8666667	
Ýnci,I50	 	 	 	D	 	 	5,2666667	
Hasanbey,I50	 	 	 	D	 	 	5,1200000	
Seçkin,I50	 	 	 	D	 	 	5,0433333	
Ýnci,I0	 	 	 	 	E	 	4,4466667	
Hasanbey,I0	 	 	 	 	 	F	4,1200000	
Seçkin,I0	 	 	 	 	 	F	4,0866667	

Levels not connected by same letter are significantly different
