# Supplementary material for: Drought stress resistance indicators of chickpea varieties grown under deficit irrigation conditions
Source: PeerJ. 2023 Mar 10;11:e14818. doi: 10.7717/peerj.14818 (PMC10010177; doi:10.7717/peerj.14818)
Supplement: Supplemental Information 3 — The positive or negative situations of the relationship between the examined characters were analyzed. It has been determined what kind of a relationship there is between the characters. [file peerj-11-14818-s003.rtf]

Multivariate 
Correlations
	verm	cwsý	cc	sul	eta	wue	nof	prd	
verm	1,0000	-0,8997	0,8773	0,7786	0,7786	-0,0684	0,9727	0,8870	
cwsý	-0,8997	1,0000	-0,9878	-0,9624	-0,9624	0,3992	-0,9115	-0,9783	
cc	0,8773	-0,9878	1,0000	0,9788	0,9788	-0,4715	0,8824	0,9870	
sul	0,7786	-0,9624	0,9788	1,0000	1,0000	-0,6011	0,7989	0,9480	
eta	0,7786	-0,9624	0,9788	1,0000	1,0000	-0,6011	0,7989	0,9480	
wue	-0,0684	0,3992	-0,4715	-0,6011	-0,6011	1,0000	-0,0823	-0,4224	
nof	0,9727	-0,9115	0,8824	0,7989	0,7989	-0,0823	1,0000	0,8938	
prd	0,8870	-0,9783	0,9870	0,9480	0,9480	-0,4224	0,8938	1,0000	

    9 rows not used due to missing values.
Scatterplot Matrix

Pairwise Correlations
Variable	by Variable	Correlation	Count	Signif Prob	Plot Corr	
cwsý	verm	-0,9406	27	0,0000		
cc	verm	0,9183	27	0,0000		
cc	cwsý	-0,9880	27	0,0000		
sul	verm	0,7786	18	0,0001		
sul	cwsý	-0,9624	18	0,0000		
sul	cc	0,9788	18	0,0000		
eta	verm	0,9196	27	0,0000		
eta	cwsý	-0,9450	27	0,0000		
eta	cc	0,9556	27	0,0000		
eta	sul	1,0000	18	0,0000		
wue	verm	-0,0015	27	0,9939		
wue	cwsý	0,2315	27	0,2453		
wue	cc	-0,3283	27	0,0945		
wue	sul	-0,6011	18	0,0083		
wue	eta	-0,3280	27	0,0949		
nof	verm	0,9880	27	0,0000		
nof	cwsý	-0,9533	27	0,0000		
nof	cc	0,9299	27	0,0000		
nof	sul	0,7989	18	0,0001		
nof	eta	0,9177	27	0,0000		
nof	wue	-0,0196	27	0,9227		
prd	verm	0,9174	27	0,0000		
prd	cwsý	-0,9831	27	0,0000		
prd	cc	0,9895	27	0,0000		
prd	sul	0,9480	18	0,0000		
prd	eta	0,9366	27	0,0000		
prd	wue	-0,2922	27	0,1391		
prd	nof	0,9335	27	0,0000		
