# Supplementary material for: Drought stress resistance indicators of chickpea varieties grown under deficit irrigation conditions
Source: PeerJ. 2023 Mar 10;11:e14818. doi: 10.7717/peerj.14818 (PMC10010177; doi:10.7717/peerj.14818)
Supplement: Supplemental Information 4 — The data obtained for each character was subjected to analysis of variance and tried to be interpreted. [file peerj-11-14818-s004.rtf]

Least Squares Fit
Response verm
Whole Model
Actual by Predicted Plot

Summary of Fit
 	 	
RSquare	0,998301	
RSquare Adj	0,996319	
Root Mean Square Error	15,15903	
Mean of Response	2171,148	
Observations (or Sum Wgts)	27	
Tests wrt Random Effects
Source	SS	MS Num	DF Num	F Ratio	Prob > F	
tek	395,63	197,815	2	0,9840	0,4492	
çeþit	229716	114858	2	571,3266	<.0001	
eþit*tek&Random	804,148	201,037	4	0,8748	0,5071	
sul konu	1376265	688132	2	2994,532	<.0001	
sul konu*eþit	13397,9	3349,48	4	14,5759	0,0001	

tek
Leverage Plot

Effect Test
Sum of Squares	F Ratio	DF	Prob > F	
395,62963	0,9840	2	0,4492	
Denominator MS Synthesis: 
 eþit*tek&Random
Least Squares Means Table
Level	Least Sq Mean	 	Std Error	Mean	
1	2172,6667		4,7262510	2172,67	
2	2165,8889		4,7262510	2165,89	
3	2174,8889		4,7262510	2174,89	

eþit
Leverage Plot

Effect Test
Sum of Squares	F Ratio	DF	Prob > F	
229715,63	571,3266	2	<.0001	
Denominator MS Synthesis: 
 eþit*tek&Random
Least Squares Means Table
Level	Least Sq Mean	 	Std Error	Mean	
Hasanbey	2186,6667		4,7262510	2186,67	
Seçkin	2051,2222		4,7262510	2051,22	
Ýnci	2275,5556		4,7262510	2275,56	
LSMeans Differences Student's t
Alpha=
0,050 t=
2,77645LSMean[i] By LSMean[j]
Mean[i]-Mean[j]
Std Err Dif
Lower CL Dif
Upper CL Dif	Hasanbey	Seçkin	Ýnci	
Hasanbey	0
0
0
0	135,444
6,68393
116,887
154,002	-88,889
6,68393
-107,45
-70,331	
Seçkin	-135,44
6,68393
-154
-116,89	0
0
0
0	-224,33
6,68393
-242,89
-205,78	
Ýnci	88,8889
6,68393
70,3313
107,446	224,333
6,68393
205,776
242,891	0
0
0
0	


Level				Least Sq Mean	
Ýnci	A	 	 	2275,5556	
Hasanbey	 	B	 	2186,6667	
Seçkin	 	 	C	2051,2222	

Levels not connected by same letter are significantly different

eþit*tek&Random
Leverage Plot

Effect Test
Sum of Squares	F Ratio	DF	Prob > F	
804,14815	0,8748	4	0,5071	
Denominator MS Synthesis: 
 Residual
Least Squares Means Table
Level	Least Sq Mean	 	Std Error	
Hasanbey,1	2180,6667		8,7520721	
Hasanbey,2	2188,0000		8,7520721	
Hasanbey,3	2191,3333		8,7520721	
Seçkin,1	2051,6667		8,7520721	
Seçkin,2	2041,6667		8,7520721	
Seçkin,3	2060,3333		8,7520721	
Ýnci,1	2285,6667		8,7520721	
Ýnci,2	2268,0000		8,7520721	
Ýnci,3	2273,0000		8,7520721	

sul konu
Leverage Plot

Effect Test
Sum of Squares	F Ratio	DF	Prob > F	
1376264,5	2994,532	2	<.0001	
Denominator MS Synthesis: 
 Residual
Least Squares Means Table
Level	Least Sq Mean	 	Std Error	Mean	
I0	1887,0000		5,0530112	1887,00	
I100	2439,3333		5,0530112	2439,33	
I50	2187,1111		5,0530112	2187,11	
LSMeans Differences Student's t
Alpha=
0,050 t=
2,17881LSMean[i] By LSMean[j]
Mean[i]-Mean[j]
Std Err Dif
Lower CL Dif
Upper CL Dif	I0	I100	I50	
I0	0
0
0
0	-552,33
7,14604
-567,9
-536,76	-300,11
7,14604
-315,68
-284,54	
I100	552,333
7,14604
536,763
567,903	0
0
0
0	252,222
7,14604
236,652
267,792	
I50	300,111
7,14604
284,541
315,681	-252,22
7,14604
-267,79
-236,65	0
0
0
0	


Level				Least Sq Mean	
I100	A	 	 	2439,3333	
I50	 	B	 	2187,1111	
I0	 	 	C	1887,0000	

Levels not connected by same letter are significantly different

sul konu*eþit
Leverage Plot

Effect Test
Sum of Squares	F Ratio	DF	Prob > F	
13397,926	14,5759	4	0,0001	
Denominator MS Synthesis: 
 Residual
Least Squares Means Table
Level	Least Sq Mean	 	Std Error	
I0,Hasanbey	1893,3333		8,7520721	
I0,Seçkin	1783,6667		8,7520721	
I0,Ýnci	1984,0000		8,7520721	
I100,Hasanbey	2423,6667		8,7520721	
I100,Seçkin	2328,0000		8,7520721	
I100,Ýnci	2566,3333		8,7520721	
I50,Hasanbey	2243,0000		8,7520721	
I50,Seçkin	2042,0000		8,7520721	
I50,Ýnci	2276,3333		8,7520721	
LSMeans Differences Student's t
Alpha=
0,050 t=
2,17881LSMean[i] By LSMean[j]
Mean[i]-Mean[j]
Std Err Dif
Lower CL Dif
Upper CL Dif	I0,Hasanbey	I0,Seçkin	I0,Ýnci	I100,Hasanbey	I100,Seçkin	I100,Ýnci	I50,Hasanbey	I50,Seçkin	I50,Ýnci	
I0,Hasanbey	0
0
0
0	109,667
12,3773
82,6988
136,634	-90,667
12,3773
-117,63
-63,699	-530,33
12,3773
-557,3
-503,37	-434,67
12,3773
-461,63
-407,7	-673
12,3773
-699,97
-646,03	-349,67
12,3773
-376,63
-322,7	-148,67
12,3773
-175,63
-121,7	-383
12,3773
-409,97
-356,03	
I0,Seçkin	-109,67
12,3773
-136,63
-82,699	0
0
0
0	-200,33
12,3773
-227,3
-173,37	-640
12,3773
-666,97
-613,03	-544,33
12,3773
-571,3
-517,37	-782,67
12,3773
-809,63
-755,7	-459,33
12,3773
-486,3
-432,37	-258,33
12,3773
-285,3
-231,37	-492,67
12,3773
-519,63
-465,7	
I0,Ýnci	90,6667
12,3773
63,6988
117,634	200,333
12,3773
173,366
227,301	0
0
0
0	-439,67
12,3773
-466,63
-412,7	-344
12,3773
-370,97
-317,03	-582,33
12,3773
-609,3
-555,37	-259
12,3773
-285,97
-232,03	-58
12,3773
-84,968
-31,032	-292,33
12,3773
-319,3
-265,37	
I100,Hasanbey	530,333
12,3773
503,366
557,301	640
12,3773
613,032
666,968	439,667
12,3773
412,699
466,634	0
0
0
0	95,6667
12,3773
68,6988
122,634	-142,67
12,3773
-169,63
-115,7	180,667
12,3773
153,699
207,634	381,667
12,3773
354,699
408,634	147,333
12,3773
120,366
174,301	
I100,Seçkin	434,667
12,3773
407,699
461,634	544,333
12,3773
517,366
571,301	344
12,3773
317,032
370,968	-95,667
12,3773
-122,63
-68,699	0
0
0
0	-238,33
12,3773
-265,3
-211,37	85
12,3773
58,0322
111,968	286
12,3773
259,032
312,968	51,6667
12,3773
24,6988
78,6345	
I100,Ýnci	673
12,3773
646,032
699,968	782,667
12,3773
755,699
809,634	582,333
12,3773
555,366
609,301	142,667
12,3773
115,699
169,634	238,333
12,3773
211,366
265,301	0
0
0
0	323,333
12,3773
296,366
350,301	524,333
12,3773
497,366
551,301	290
12,3773
263,032
316,968	
I50,Hasanbey	349,667
12,3773
322,699
376,634	459,333
12,3773
432,366
486,301	259
12,3773
232,032
285,968	-180,67
12,3773
-207,63
-153,7	-85
12,3773
-111,97
-58,032	-323,33
12,3773
-350,3
-296,37	0
0
0
0	201
12,3773
174,032
227,968	-33,333
12,3773
-60,301
-6,3655	
I50,Seçkin	148,667
12,3773
121,699
175,634	258,333
12,3773
231,366
285,301	58
12,3773
31,0322
84,9678	-381,67
12,3773
-408,63
-354,7	-286
12,3773
-312,97
-259,03	-524,33
12,3773
-551,3
-497,37	-201
12,3773
-227,97
-174,03	0
0
0
0	-234,33
12,3773
-261,3
-207,37	
I50,Ýnci	383
12,3773
356,032
409,968	492,667
12,3773
465,699
519,634	292,333
12,3773
265,366
319,301	-147,33
12,3773
-174,3
-120,37	-51,667
12,3773
-78,634
-24,699	-290
12,3773
-316,97
-263,03	33,3333
12,3773
6,36552
60,3012	234,333
12,3773
207,366
261,301	0
0
0
0	


Level										Least Sq Mean	
I100,Ýnci	A	 	 	 	 	 	 	 	 	2566,3333	
I100,Hasanbey	 	B	 	 	 	 	 	 	 	2423,6667	
I100,Seçkin	 	 	C	 	 	 	 	 	 	2328,0000	
I50,Ýnci	 	 	 	D	 	 	 	 	 	2276,3333	
I50,Hasanbey	 	 	 	 	E	 	 	 	 	2243,0000	
I50,Seçkin	 	 	 	 	 	F	 	 	 	2042,0000	
I0,Ýnci	 	 	 	 	 	 	G	 	 	1984,0000	
I0,Hasanbey	 	 	 	 	 	 	 	H	 	1893,3333	
I0,Seçkin	 	 	 	 	 	 	 	 	I	1783,6667	

Levels not connected by same letter are significantly different
Response cwsý
Whole Model
Summary of Fit
 	 	
RSquare	0,997125	
RSquare Adj	0,993771	
Root Mean Square Error	0,010887	
Mean of Response	0,40963	
Observations (or Sum Wgts)	27	
Tests wrt Random Effects
Source	SS	MS Num	DF Num	F Ratio	Prob > F	
tek	0,0001	0,00005	2	0,6842	0,5552	
eþit	0,02525	0,01263	2	179,4211	0,0001	
eþit*tek&Random	0,00028	0,00007	4	0,5937	0,6739	
sul konu	0,46759	0,23379	2	1972,625	<.0001	
sul konu*eþit	0,00006	0,00001	4	0,1250	0,9706	

tek
Leverage Plot

Effect Test
Sum of Squares	F Ratio	DF	Prob > F	
0,00009630	0,6842	2	0,5552	
Denominator MS Synthesis: 
 eþit*tek&Random
Least Squares Means Table
Level	Least Sq Mean	 	Std Error	Mean	
1	0,40777778		0,00279623	0,407778	
2	0,40888889		0,00279623	0,408889	
3	0,41222222		0,00279623	0,412222	

eþit
Leverage Plot

Effect Test
Sum of Squares	F Ratio	DF	Prob > F	
0,02525185	179,4211	2	0,0001	
Denominator MS Synthesis: 
 eþit*tek&Random
Least Squares Means Table
Level	Least Sq Mean	 	Std Error	Mean	
Hasanbey	0,41444444		0,00279623	0,414444	
Seçkin	0,44444444		0,00279623	0,444444	
Ýnci	0,37000000		0,00279623	0,370000	
LSMeans Differences Student's t
Alpha=
0,050 t=
2,77645LSMean[i] By LSMean[j]
Mean[i]-Mean[j]
Std Err Dif
Lower CL Dif
Upper CL Dif	Hasanbey	Seçkin	Ýnci	
Hasanbey	0
0
0
0	-0,03
0,00395
-0,041
-0,019	0,04444
0,00395
0,03347
0,05542	
Seçkin	0,03
0,00395
0,01902
0,04098	0
0
0
0	0,07444
0,00395
0,06347
0,08542	
Ýnci	-0,0444
0,00395
-0,0554
-0,0335	-0,0744
0,00395
-0,0854
-0,0635	0
0
0
0	


Level				Least Sq Mean	
Seçkin	A	 	 	0,44444444	
Hasanbey	 	B	 	0,41444444	
Ýnci	 	 	C	0,37000000	

Levels not connected by same letter are significantly different

eþit*tek&Random
Leverage Plot

Effect Test
Sum of Squares	F Ratio	DF	Prob > F	
0,00028148	0,5937	4	0,6739	
Denominator MS Synthesis: 
 Residual
Least Squares Means Table
Level	Least Sq Mean	 	Std Error	
Hasanbey,1	0,41000000		0,00628539	
Hasanbey,2	0,42000000		0,00628539	
Hasanbey,3	0,41333333		0,00628539	
Seçkin,1	0,44333333		0,00628539	
Seçkin,2	0,44000000		0,00628539	
Seçkin,3	0,45000000		0,00628539	
Ýnci,1	0,37000000		0,00628539	
Ýnci,2	0,36666667		0,00628539	
Ýnci,3	0,37333333		0,00628539	

sul konu
Leverage Plot

Effect Test
Sum of Squares	F Ratio	DF	Prob > F	
0,46758519	1972,625	2	<.0001	
Denominator MS Synthesis: 
 Residual
Least Squares Means Table
Level	Least Sq Mean	 	Std Error	Mean	
I0	0,54444444		0,00362887	0,544444	
I100	0,23111111		0,00362887	0,231111	
I50	0,45333333		0,00362887	0,453333	
LSMeans Differences Student's t
Alpha=
0,050 t=
2,17881LSMean[i] By LSMean[j]
Mean[i]-Mean[j]
Std Err Dif
Lower CL Dif
Upper CL Dif	I0	I100	I50	
I0	0
0
0
0	0,31333
0,00513
0,30215
0,32452	0,09111
0,00513
0,07993
0,10229	
I100	-0,3133
0,00513
-0,3245
-0,3022	0
0
0
0	-0,2222
0,00513
-0,2334
-0,211	
I50	-0,0911
0,00513
-0,1023
-0,0799	0,22222
0,00513
0,21104
0,2334	0
0
0
0	


Level				Least Sq Mean	
I0	A	 	 	0,54444444	
I50	 	B	 	0,45333333	
I100	 	 	C	0,23111111	

Levels not connected by same letter are significantly different

sul konu*eþit
Leverage Plot

Effect Test
Sum of Squares	F Ratio	DF	Prob > F	
0,00005926	0,1250	4	0,9706	
Denominator MS Synthesis: 
 Residual
Least Squares Means Table
Level	Least Sq Mean	 	Std Error	
I0,Hasanbey	0,55000000		0,00628539	
I0,Seçkin	0,58000000		0,00628539	
I0,Ýnci	0,50333333		0,00628539	
I100,Hasanbey	0,23333333		0,00628539	
I100,Seçkin	0,26666667		0,00628539	
I100,Ýnci	0,19333333		0,00628539	
I50,Hasanbey	0,46000000		0,00628539	
I50,Seçkin	0,48666667		0,00628539	
I50,Ýnci	0,41333333		0,00628539	
LSMeans Differences Student's t
Alpha=
0,050 t=
2,17881LSMean[i] By LSMean[j]
Mean[i]-Mean[j]
Std Err Dif
Lower CL Dif
Upper CL Dif	I0,Hasanbey	I0,Seçkin	I0,Ýnci	I100,Hasanbey	I100,Seçkin	I100,Ýnci	I50,Hasanbey	I50,Seçkin	I50,Ýnci	
I0,Hasanbey	0
0
0
0	-0,03
0,00889
-0,0494
-0,0106	0,04667
0,00889
0,0273
0,06603	0,31667
0,00889
0,2973
0,33603	0,28333
0,00889
0,26397
0,3027	0,35667
0,00889
0,3373
0,37603	0,09
0,00889
0,07063
0,10937	0,06333
0,00889
0,04397
0,0827	0,13667
0,00889
0,1173
0,15603	
I0,Seçkin	0,03
0,00889
0,01063
0,04937	0
0
0
0	0,07667
0,00889
0,0573
0,09603	0,34667
0,00889
0,3273
0,36603	0,31333
0,00889
0,29397
0,3327	0,38667
0,00889
0,3673
0,40603	0,12
0,00889
0,10063
0,13937	0,09333
0,00889
0,07397
0,1127	0,16667
0,00889
0,1473
0,18603	
I0,Ýnci	-0,0467
0,00889
-0,066
-0,0273	-0,0767
0,00889
-0,096
-0,0573	0
0
0
0	0,27
0,00889
0,25063
0,28937	0,23667
0,00889
0,2173
0,25603	0,31
0,00889
0,29063
0,32937	0,04333
0,00889
0,02397
0,0627	0,01667
0,00889
-0,0027
0,03603	0,09
0,00889
0,07063
0,10937	
I100,Hasanbey	-0,3167
0,00889
-0,336
-0,2973	-0,3467
0,00889
-0,366
-0,3273	-0,27
0,00889
-0,2894
-0,2506	0
0
0
0	-0,0333
0,00889
-0,0527
-0,014	0,04
0,00889
0,02063
0,05937	-0,2267
0,00889
-0,246
-0,2073	-0,2533
0,00889
-0,2727
-0,234	-0,18
0,00889
-0,1994
-0,1606	
I100,Seçkin	-0,2833
0,00889
-0,3027
-0,264	-0,3133
0,00889
-0,3327
-0,294	-0,2367
0,00889
-0,256
-0,2173	0,03333
0,00889
0,01397
0,0527	0
0
0
0	0,07333
0,00889
0,05397
0,0927	-0,1933
0,00889
-0,2127
-0,174	-0,22
0,00889
-0,2394
-0,2006	-0,1467
0,00889
-0,166
-0,1273	
I100,Ýnci	-0,3567
0,00889
-0,376
-0,3373	-0,3867
0,00889
-0,406
-0,3673	-0,31
0,00889
-0,3294
-0,2906	-0,04
0,00889
-0,0594
-0,0206	-0,0733
0,00889
-0,0927
-0,054	0
0
0
0	-0,2667
0,00889
-0,286
-0,2473	-0,2933
0,00889
-0,3127
-0,274	-0,22
0,00889
-0,2394
-0,2006	
I50,Hasanbey	-0,09
0,00889
-0,1094
-0,0706	-0,12
0,00889
-0,1394
-0,1006	-0,0433
0,00889
-0,0627
-0,024	0,22667
0,00889
0,2073
0,24603	0,19333
0,00889
0,17397
0,2127	0,26667
0,00889
0,2473
0,28603	0
0
0
0	-0,0267
0,00889
-0,046
-0,0073	0,04667
0,00889
0,0273
0,06603	
I50,Seçkin	-0,0633
0,00889
-0,0827
-0,044	-0,0933
0,00889
-0,1127
-0,074	-0,0167
0,00889
-0,036
0,0027	0,25333
0,00889
0,23397
0,2727	0,22
0,00889
0,20063
0,23937	0,29333
0,00889
0,27397
0,3127	0,02667
0,00889
0,0073
0,04603	0
0
0
0	0,07333
0,00889
0,05397
0,0927	
I50,Ýnci	-0,1367
0,00889
-0,156
-0,1173	-0,1667
0,00889
-0,186
-0,1473	-0,09
0,00889
-0,1094
-0,0706	0,18
0,00889
0,16063
0,19937	0,14667
0,00889
0,1273
0,16603	0,22
0,00889
0,20063
0,23937	-0,0467
0,00889
-0,066
-0,0273	-0,0733
0,00889
-0,0927
-0,054	0
0
0
0	


Level									Least Sq Mean	
I0,Seçkin	A	 	 	 	 	 	 	 	0,58000000	
I0,Hasanbey	 	B	 	 	 	 	 	 	0,55000000	
I0,Ýnci	 	 	C	 	 	 	 	 	0,50333333	
I50,Seçkin	 	 	C	 	 	 	 	 	0,48666667	
I50,Hasanbey	 	 	 	D	 	 	 	 	0,46000000	
I50,Ýnci	 	 	 	 	E	 	 	 	0,41333333	
I100,Seçkin	 	 	 	 	 	F	 	 	0,26666667	
I100,Hasanbey	 	 	 	 	 	 	G	 	0,23333333	
I100,Ýnci	 	 	 	 	 	 	 	H	0,19333333	

Levels not connected by same letter are significantly different
Response cc
Whole Model
Summary of Fit
 	 	
RSquare	0,99831	
RSquare Adj	0,996338	
Root Mean Square Error	0,269588	
Mean of Response	37,45778	
Observations (or Sum Wgts)	27	
Tests wrt Random Effects
Source	SS	MS Num	DF Num	F Ratio	Prob > F	
tek	0,27129	0,13564	2	0,8689	0,4860	
eþit	8,55127	4,27563	2	27,3884	0,0046	
eþit*tek&Random	0,62444	0,15611	4	2,1480	0,1372	
sul konu	503,113	251,557	2	3461,258	<.0001	
sul konu*eþit	2,54244	0,63561	4	8,7456	0,0015	
Residual by Predicted Plot


tek
Leverage Plot

Effect Test
Sum of Squares	F Ratio	DF	Prob > F	
0,27128889	0,8689	2	0,4860	
Denominator MS Synthesis: 
 eþit*tek&Random
Least Squares Means Table
Level	Least Sq Mean	 	Std Error	Mean	
1	37,471111		0,13170300	37,4711	
2	37,328889		0,13170300	37,3289	
3	37,573333		0,13170300	37,5733	

eþit
Leverage Plot

Effect Test
Sum of Squares	F Ratio	DF	Prob > F	
8,5512667	27,3884	2	0,0046	
Denominator MS Synthesis: 
 eþit*tek&Random
Least Squares Means Table
Level	Least Sq Mean	 	Std Error	Mean	
Hasanbey	37,498889		0,13170300	37,4989	
Seçkin	36,748889		0,13170300	36,7489	
Ýnci	38,125556		0,13170300	38,1256	
LSMeans Differences Student's t
Alpha=
0,050 t=
2,77645LSMean[i] By LSMean[j]
Mean[i]-Mean[j]
Std Err Dif
Lower CL Dif
Upper CL Dif	Hasanbey	Seçkin	Ýnci	
Hasanbey	0
0
0
0	0,75
0,18626
0,23287
1,26713	-0,6267
0,18626
-1,1438
-0,1095	
Seçkin	-0,75
0,18626
-1,2671
-0,2329	0
0
0
0	-1,3767
0,18626
-1,8938
-0,8595	
Ýnci	0,62667
0,18626
0,10954
1,1438	1,37667
0,18626
0,85954
1,8938	0
0
0
0	


Level				Least Sq Mean	
Ýnci	A	 	 	38,125556	
Hasanbey	 	B	 	37,498889	
Seçkin	 	 	C	36,748889	

Levels not connected by same letter are significantly different

eþit*tek&Random
Leverage Plot

Effect Test
Sum of Squares	F Ratio	DF	Prob > F	
0,62444444	2,1480	4	0,1372	
Denominator MS Synthesis: 
 Residual
Least Squares Means Table
Level	Least Sq Mean	 	Std Error	
Hasanbey,1	37,590000		0,15564680	
Hasanbey,2	37,316667		0,15564680	
Hasanbey,3	37,590000		0,15564680	
Seçkin,1	36,546667		0,15564680	
Seçkin,2	36,893333		0,15564680	
Seçkin,3	36,806667		0,15564680	
Ýnci,1	38,276667		0,15564680	
Ýnci,2	37,776667		0,15564680	
Ýnci,3	38,323333		0,15564680	

sul konu
Leverage Plot

Effect Test
Sum of Squares	F Ratio	DF	Prob > F	
503,11309	3461,258	2	<.0001	
Denominator MS Synthesis: 
 Residual
Least Squares Means Table
Level	Least Sq Mean	 	Std Error	Mean	
I0	33,111111		0,08986272	33,1111	
I100	43,343333		0,08986272	43,3433	
I50	35,918889		0,08986272	35,9189	
LSMeans Differences Student's t
Alpha=
0,050 t=
2,17881LSMean[i] By LSMean[j]
Mean[i]-Mean[j]
Std Err Dif
Lower CL Dif
Upper CL Dif	I0	I100	I50	
I0	0
0
0
0	-10,232
0,12709
-10,509
-9,9553	-2,8078
0,12709
-3,0847
-2,5309	
I100	10,2322
0,12709
9,95533
10,5091	0
0
0
0	7,42444
0,12709
7,14755
7,70134	
I50	2,80778
0,12709
2,53088
3,08467	-7,4244
0,12709
-7,7013
-7,1475	0
0
0
0	


Level				Least Sq Mean	
I100	A	 	 	43,343333	
I50	 	B	 	35,918889	
I0	 	 	C	33,111111	

Levels not connected by same letter are significantly different

sul konu*eþit
Leverage Plot

Effect Test
Sum of Squares	F Ratio	DF	Prob > F	
2,5424444	8,7456	4	0,0015	
Denominator MS Synthesis: 
 Residual
Least Squares Means Table
Level	Least Sq Mean	 	Std Error	
I0,Hasanbey	32,850000		0,15564680	
I0,Seçkin	32,880000		0,15564680	
I0,Ýnci	33,603333		0,15564680	
I100,Hasanbey	43,520000		0,15564680	
I100,Seçkin	42,120000		0,15564680	
I100,Ýnci	44,390000		0,15564680	
I50,Hasanbey	36,126667		0,15564680	
I50,Seçkin	35,246667		0,15564680	
I50,Ýnci	36,383333		0,15564680	
LSMeans Differences Student's t
Alpha=
0,050 t=
2,17881LSMean[i] By LSMean[j]
Mean[i]-Mean[j]
Std Err Dif
Lower CL Dif
Upper CL Dif	I0,Hasanbey	I0,Seçkin	I0,Ýnci	I100,Hasanbey	I100,Seçkin	I100,Ýnci	I50,Hasanbey	I50,Seçkin	I50,Ýnci	
I0,Hasanbey	0
0
0
0	-0,03
0,22012
-0,5096
0,4496	-0,7533
0,22012
-1,2329
-0,2737	-10,67
0,22012
-11,15
-10,19	-9,27
0,22012
-9,7496
-8,7904	-11,54
0,22012
-12,02
-11,06	-3,2767
0,22012
-3,7563
-2,7971	-2,3967
0,22012
-2,8763
-1,9171	-3,5333
0,22012
-4,0129
-3,0537	
I0,Seçkin	0,03
0,22012
-0,4496
0,5096	0
0
0
0	-0,7233
0,22012
-1,2029
-0,2437	-10,64
0,22012
-11,12
-10,16	-9,24
0,22012
-9,7196
-8,7604	-11,51
0,22012
-11,99
-11,03	-3,2467
0,22012
-3,7263
-2,7671	-2,3667
0,22012
-2,8463
-1,8871	-3,5033
0,22012
-3,9829
-3,0237	
I0,Ýnci	0,75333
0,22012
0,27374
1,23293	0,72333
0,22012
0,24374
1,20293	0
0
0
0	-9,9167
0,22012
-10,396
-9,4371	-8,5167
0,22012
-8,9963
-8,0371	-10,787
0,22012
-11,266
-10,307	-2,5233
0,22012
-3,0029
-2,0437	-1,6433
0,22012
-2,1229
-1,1637	-2,78
0,22012
-3,2596
-2,3004	
I100,Hasanbey	10,67
0,22012
10,1904
11,1496	10,64
0,22012
10,1604
11,1196	9,91667
0,22012
9,43707
10,3963	0
0
0
0	1,4
0,22012
0,9204
1,8796	-0,87
0,22012
-1,3496
-0,3904	7,39333
0,22012
6,91374
7,87293	8,27333
0,22012
7,79374
8,75293	7,13667
0,22012
6,65707
7,61626	
I100,Seçkin	9,27
0,22012
8,7904
9,7496	9,24
0,22012
8,7604
9,7196	8,51667
0,22012
8,03707
8,99626	-1,4
0,22012
-1,8796
-0,9204	0
0
0
0	-2,27
0,22012
-2,7496
-1,7904	5,99333
0,22012
5,51374
6,47293	6,87333
0,22012
6,39374
7,35293	5,73667
0,22012
5,25707
6,21626	
I100,Ýnci	11,54
0,22012
11,0604
12,0196	11,51
0,22012
11,0304
11,9896	10,7867
0,22012
10,3071
11,2663	0,87
0,22012
0,3904
1,3496	2,27
0,22012
1,7904
2,7496	0
0
0
0	8,26333
0,22012
7,78374
8,74293	9,14333
0,22012
8,66374
9,62293	8,00667
0,22012
7,52707
8,48626	
I50,Hasanbey	3,27667
0,22012
2,79707
3,75626	3,24667
0,22012
2,76707
3,72626	2,52333
0,22012
2,04374
3,00293	-7,3933
0,22012
-7,8729
-6,9137	-5,9933
0,22012
-6,4729
-5,5137	-8,2633
0,22012
-8,7429
-7,7837	0
0
0
0	0,88
0,22012
0,4004
1,3596	-0,2567
0,22012
-0,7363
0,22293	
I50,Seçkin	2,39667
0,22012
1,91707
2,87626	2,36667
0,22012
1,88707
2,84626	1,64333
0,22012
1,16374
2,12293	-8,2733
0,22012
-8,7529
-7,7937	-6,8733
0,22012
-7,3529
-6,3937	-9,1433
0,22012
-9,6229
-8,6637	-0,88
0,22012
-1,3596
-0,4004	0
0
0
0	-1,1367
0,22012
-1,6163
-0,6571	
I50,Ýnci	3,53333
0,22012
3,05374
4,01293	3,50333
0,22012
3,02374
3,98293	2,78
0,22012
2,3004
3,2596	-7,1367
0,22012
-7,6163
-6,6571	-5,7367
0,22012
-6,2163
-5,2571	-8,0067
0,22012
-8,4863
-7,5271	0,25667
0,22012
-0,2229
0,73626	1,13667
0,22012
0,65707
1,61626	0
0
0
0	


Level								Least Sq Mean	
I100,Ýnci	A	 	 	 	 	 	 	44,390000	
I100,Hasanbey	 	B	 	 	 	 	 	43,520000	
I100,Seçkin	 	 	C	 	 	 	 	42,120000	
I50,Ýnci	 	 	 	D	 	 	 	36,383333	
I50,Hasanbey	 	 	 	D	 	 	 	36,126667	
I50,Seçkin	 	 	 	 	E	 	 	35,246667	
I0,Ýnci	 	 	 	 	 	F	 	33,603333	
I0,Seçkin	 	 	 	 	 	 	G	32,880000	
I0,Hasanbey	 	 	 	 	 	 	G	32,850000	

Levels not connected by same letter are significantly different
Response wue
Whole Model
Summary of Fit
 	 	
RSquare	0,913932	
RSquare Adj	0,813518	
Root Mean Square Error	0,015215	
Mean of Response	0,595185	
Observations (or Sum Wgts)	27	
Tests wrt Random Effects
Source	SS	MS Num	DF Num	F Ratio	Prob > F	
tek	0,00012	0,00006	2	1,0000	0,4444	
eþit	0,01939	0,00969	2	163,5625	0,0001	
eþit*tek&Random	0,00024	0,00006	4	0,2560	0,9004	
sul konu	0,00907	0,00454	2	19,6000	0,0002	
sul konu*eþit	0,00068	0,00017	4	0,7360	0,5850	

tek
Leverage Plot

Effect Test
Sum of Squares	F Ratio	DF	Prob > F	
0,00011852	1,0000	2	0,4444	
Denominator MS Synthesis: 
 eþit*tek&Random
Least Squares Means Table
Level	Least Sq Mean	 	Std Error	Mean	
1	0,59222222		0,00256600	0,592222	
2	0,59666667		0,00256600	0,596667	
3	0,59666667		0,00256600	0,596667	

eþit
Leverage Plot

Effect Test
Sum of Squares	F Ratio	DF	Prob > F	
0,01938519	163,5625	2	0,0001	
Denominator MS Synthesis: 
 eþit*tek&Random
Least Squares Means Table
Level	Least Sq Mean	 	Std Error	Mean	
Hasanbey	0,59333333		0,00256600	0,593333	
Seçkin	0,56333333		0,00256600	0,563333	
Ýnci	0,62888889		0,00256600	0,628889	
LSMeans Differences Student's t
Alpha=
0,050 t=
2,77645LSMean[i] By LSMean[j]
Mean[i]-Mean[j]
Std Err Dif
Lower CL Dif
Upper CL Dif	Hasanbey	Seçkin	Ýnci	
Hasanbey	0
0
0
0	0,03
0,00363
0,01992
0,04008	-0,0356
0,00363
-0,0456
-0,0255	
Seçkin	-0,03
0,00363
-0,0401
-0,0199	0
0
0
0	-0,0656
0,00363
-0,0756
-0,0555	
Ýnci	0,03556
0,00363
0,02548
0,04563	0,06556
0,00363
0,05548
0,07563	0
0
0
0	


Level				Least Sq Mean	
Ýnci	A	 	 	0,62888889	
Hasanbey	 	B	 	0,59333333	
Seçkin	 	 	C	0,56333333	

Levels not connected by same letter are significantly different

eþit*tek&Random
Leverage Plot

Effect Test
Sum of Squares	F Ratio	DF	Prob > F	
0,00023704	0,2560	4	0,9004	
Denominator MS Synthesis: 
 Residual
Least Squares Means Table
Level	Least Sq Mean	 	Std Error	
Hasanbey,1	0,59333333		0,00878410	
Hasanbey,2	0,59333333		0,00878410	
Hasanbey,3	0,59333333		0,00878410	
Seçkin,1	0,55666667		0,00878410	
Seçkin,2	0,56333333		0,00878410	
Seçkin,3	0,57000000		0,00878410	
Ýnci,1	0,62666667		0,00878410	
Ýnci,2	0,63333333		0,00878410	
Ýnci,3	0,62666667		0,00878410	

sul konu
Leverage Plot

Effect Test
Sum of Squares	F Ratio	DF	Prob > F	
0,00907407	19,6000	2	0,0002	
Denominator MS Synthesis: 
 Residual
Least Squares Means Table
Level	Least Sq Mean	 	Std Error	Mean	
I0	0,59888889		0,00507151	0,598889	
I100	0,57111111		0,00507151	0,571111	
I50	0,61555556		0,00507151	0,615556	
LSMeans Differences Student's t
Alpha=
0,050 t=
2,17881LSMean[i] By LSMean[j]
Mean[i]-Mean[j]
Std Err Dif
Lower CL Dif
Upper CL Dif	I0	I100	I50	
I0	0
0
0
0	0,02778
0,00717
0,01215
0,0434	-0,0167
0,00717
-0,0323
-0,001	
I100	-0,0278
0,00717
-0,0434
-0,0122	0
0
0
0	-0,0444
0,00717
-0,0601
-0,0288	
I50	0,01667
0,00717
0,00104
0,03229	0,04444
0,00717
0,02882
0,06007	0
0
0
0	


Level				Least Sq Mean	
I50	A	 	 	0,61555556	
I0	 	B	 	0,59888889	
I100	 	 	C	0,57111111	

Levels not connected by same letter are significantly different

sul konu*eþit
Leverage Plot

Effect Test
Sum of Squares	F Ratio	DF	Prob > F	
0,00068148	0,7360	4	0,5850	
Denominator MS Synthesis: 
 Residual
Least Squares Means Table
Level	Least Sq Mean	 	Std Error	
I0,Hasanbey	0,60000000		0,00878410	
I0,Seçkin	0,56333333		0,00878410	
I0,Ýnci	0,63333333		0,00878410	
I100,Hasanbey	0,57000000		0,00878410	
I100,Seçkin	0,54666667		0,00878410	
I100,Ýnci	0,59666667		0,00878410	
I50,Hasanbey	0,61000000		0,00878410	
I50,Seçkin	0,58000000		0,00878410	
I50,Ýnci	0,65666667		0,00878410	
LSMeans Differences Student's t
Alpha=
0,050 t=
2,17881LSMean[i] By LSMean[j]
Mean[i]-Mean[j]
Std Err Dif
Lower CL Dif
Upper CL Dif	I0,Hasanbey	I0,Seçkin	I0,Ýnci	I100,Hasanbey	I100,Seçkin	I100,Ýnci	I50,Hasanbey	I50,Seçkin	I50,Ýnci	
I0,Hasanbey	0
0
0
0	0,03667
0,01242
0,0096
0,06373	-0,0333
0,01242
-0,0604
-0,0063	0,03
0,01242
0,00293
0,05707	0,05333
0,01242
0,02627
0,0804	0,00333
0,01242
-0,0237
0,0304	-0,01
0,01242
-0,0371
0,01707	0,02
0,01242
-0,0071
0,04707	-0,0567
0,01242
-0,0837
-0,0296	
I0,Seçkin	-0,0367
0,01242
-0,0637
-0,0096	0
0
0
0	-0,07
0,01242
-0,0971
-0,0429	-0,0067
0,01242
-0,0337
0,0204	0,01667
0,01242
-0,0104
0,04373	-0,0333
0,01242
-0,0604
-0,0063	-0,0467
0,01242
-0,0737
-0,0196	-0,0167
0,01242
-0,0437
0,0104	-0,0933
0,01242
-0,1204
-0,0663	
I0,Ýnci	0,03333
0,01242
0,00627
0,0604	0,07
0,01242
0,04293
0,09707	0
0
0
0	0,06333
0,01242
0,03627
0,0904	0,08667
0,01242
0,0596
0,11373	0,03667
0,01242
0,0096
0,06373	0,02333
0,01242
-0,0037
0,0504	0,05333
0,01242
0,02627
0,0804	-0,0233
0,01242
-0,0504
0,00373	
I100,Hasanbey	-0,03
0,01242
-0,0571
-0,0029	0,00667
0,01242
-0,0204
0,03373	-0,0633
0,01242
-0,0904
-0,0363	0
0
0
0	0,02333
0,01242
-0,0037
0,0504	-0,0267
0,01242
-0,0537
0,0004	-0,04
0,01242
-0,0671
-0,0129	-0,01
0,01242
-0,0371
0,01707	-0,0867
0,01242
-0,1137
-0,0596	
I100,Seçkin	-0,0533
0,01242
-0,0804
-0,0263	-0,0167
0,01242
-0,0437
0,0104	-0,0867
0,01242
-0,1137
-0,0596	-0,0233
0,01242
-0,0504
0,00373	0
0
0
0	-0,05
0,01242
-0,0771
-0,0229	-0,0633
0,01242
-0,0904
-0,0363	-0,0333
0,01242
-0,0604
-0,0063	-0,11
0,01242
-0,1371
-0,0829	
I100,Ýnci	-0,0033
0,01242
-0,0304
0,02373	0,03333
0,01242
0,00627
0,0604	-0,0367
0,01242
-0,0637
-0,0096	0,02667
0,01242
-0,0004
0,05373	0,05
0,01242
0,02293
0,07707	0
0
0
0	-0,0133
0,01242
-0,0404
0,01373	0,01667
0,01242
-0,0104
0,04373	-0,06
0,01242
-0,0871
-0,0329	
I50,Hasanbey	0,01
0,01242
-0,0171
0,03707	0,04667
0,01242
0,0196
0,07373	-0,0233
0,01242
-0,0504
0,00373	0,04
0,01242
0,01293
0,06707	0,06333
0,01242
0,03627
0,0904	0,01333
0,01242
-0,0137
0,0404	0
0
0
0	0,03
0,01242
0,00293
0,05707	-0,0467
0,01242
-0,0737
-0,0196	
I50,Seçkin	-0,02
0,01242
-0,0471
0,00707	0,01667
0,01242
-0,0104
0,04373	-0,0533
0,01242
-0,0804
-0,0263	0,01
0,01242
-0,0171
0,03707	0,03333
0,01242
0,00627
0,0604	-0,0167
0,01242
-0,0437
0,0104	-0,03
0,01242
-0,0571
-0,0029	0
0
0
0	-0,0767
0,01242
-0,1037
-0,0496	
I50,Ýnci	0,05667
0,01242
0,0296
0,08373	0,09333
0,01242
0,06627
0,1204	0,02333
0,01242
-0,0037
0,0504	0,08667
0,01242
0,0596
0,11373	0,11
0,01242
0,08293
0,13707	0,06
0,01242
0,03293
0,08707	0,04667
0,01242
0,0196
0,07373	0,07667
0,01242
0,0496
0,10373	0
0
0
0	


Level								Least Sq Mean	
I50,Ýnci	A	 	 	 	 	 	 	0,65666667	
I0,Ýnci	A	B	 	 	 	 	 	0,63333333	
I50,Hasanbey	 	B	C	 	 	 	 	0,61000000	
I0,Hasanbey	 	 	C	D	 	 	 	0,60000000	
I100,Ýnci	 	 	C	D	E	 	 	0,59666667	
I50,Seçkin	 	 	 	D	E	F	 	0,58000000	
I100,Hasanbey	 	 	 	 	E	F	G	0,57000000	
I0,Seçkin	 	 	 	 	 	F	G	0,56333333	
I100,Seçkin	 	 	 	 	 	 	G	0,54666667	

Levels not connected by same letter are significantly different
Response nodul
Whole Model
Summary of Fit
 	 	
RSquare	0,993569	
RSquare Adj	0,986066	
Root Mean Square Error	0,174738	
Mean of Response	8,562593	
Observations (or Sum Wgts)	27	
Tests wrt Random Effects
Source	SS	MS Num	DF Num	F Ratio	Prob > F	
tek	0,0065	0,00325	2	0,2868	0,7649	
eþit	7,78892	3,89446	2	343,8535	<.0001	
eþit*tek&Random	0,0453	0,01133	4	0,3709	0,8249	
sul konu	48,0007	24,0004	2	786,0380	<.0001	
sul konu*eþit	0,76668	0,19167	4	6,2774	0,0058	
Residual by Predicted Plot


tek
Leverage Plot

Effect Test
Sum of Squares	F Ratio	DF	Prob > F	
0,00649630	0,2868	2	0,7649	
Denominator MS Synthesis: 
 eþit*tek&Random
Least Squares Means Table
Level	Least Sq Mean	 	Std Error	Mean	
1	8,5500000		0,03547444	8,55000	
2	8,5533333		0,03547444	8,55333	
3	8,5844444		0,03547444	8,58444	

eþit
Leverage Plot

Effect Test
Sum of Squares	F Ratio	DF	Prob > F	
7,7889185	343,8535	2	<.0001	
Denominator MS Synthesis: 
 eþit*tek&Random
Least Squares Means Table
Level	Least Sq Mean	 	Std Error	Mean	
Hasanbey	8,4544444		0,03547444	8,45444	
Seçkin	7,9655556		0,03547444	7,96556	
Ýnci	9,2677778		0,03547444	9,26778	
LSMeans Differences Student's t
Alpha=
0,050 t=
2,77645LSMean[i] By LSMean[j]
Mean[i]-Mean[j]
Std Err Dif
Lower CL Dif
Upper CL Dif	Hasanbey	Seçkin	Ýnci	
Hasanbey	0
0
0
0	0,48889
0,05017
0,3496
0,62818	-0,8133
0,05017
-0,9526
-0,674	
Seçkin	-0,4889
0,05017
-0,6282
-0,3496	0
0
0
0	-1,3022
0,05017
-1,4415
-1,1629	
Ýnci	0,81333
0,05017
0,67404
0,95262	1,30222
0,05017
1,16293
1,44151	0
0
0
0	


Level				Least Sq Mean	
Ýnci	A	 	 	9,2677778	
Hasanbey	 	B	 	8,4544444	
Seçkin	 	 	C	7,9655556	

Levels not connected by same letter are significantly different

eþit*tek&Random
Leverage Plot

Effect Test
Sum of Squares	F Ratio	DF	Prob > F	
0,04530370	0,3709	4	0,8249	
Denominator MS Synthesis: 
 Residual
Least Squares Means Table
Level	Least Sq Mean	 	Std Error	
Hasanbey,1	8,4366667		0,10088497	
Hasanbey,2	8,4733333		0,10088497	
Hasanbey,3	8,4533333		0,10088497	
Seçkin,1	7,9033333		0,10088497	
Seçkin,2	7,9333333		0,10088497	
Seçkin,3	8,0600000		0,10088497	
Ýnci,1	9,3100000		0,10088497	
Ýnci,2	9,2533333		0,10088497	
Ýnci,3	9,2400000		0,10088497	

sul konu
Leverage Plot

Effect Test
Sum of Squares	F Ratio	DF	Prob > F	
48,000719	786,0380	2	<.0001	
Denominator MS Synthesis: 
 Residual
Least Squares Means Table
Level	Least Sq Mean	 	Std Error	Mean	
I0	6,945556		0,05824597	6,9456	
I100	10,211111		0,05824597	10,2111	
I50	8,531111		0,05824597	8,5311	
LSMeans Differences Student's t
Alpha=
0,050 t=
2,17881LSMean[i] By LSMean[j]
Mean[i]-Mean[j]
Std Err Dif
Lower CL Dif
Upper CL Dif	I0	I100	I50	
I0	0
0
0
0	-3,2656
0,08237
-3,445
-3,0861	-1,5856
0,08237
-1,765
-1,4061	
I100	3,26556
0,08237
3,08608
3,44503	0
0
0
0	1,68
0,08237
1,50053
1,85947	
I50	1,58556
0,08237
1,40608
1,76503	-1,68
0,08237
-1,8595
-1,5005	0
0
0
0	


Level				Least Sq Mean	
I100	A	 	 	10,211111	
I50	 	B	 	8,531111	
I0	 	 	C	6,945556	

Levels not connected by same letter are significantly different

sul konu*eþit
Leverage Plot

Effect Test
Sum of Squares	F Ratio	DF	Prob > F	
0,76668148	6,2774	4	0,0058	
Denominator MS Synthesis: 
 Residual
Least Squares Means Table
Level	Least Sq Mean	 	Std Error	
I0,Hasanbey	6,880000		0,10088497	
I0,Seçkin	6,443333		0,10088497	
I0,Ýnci	7,513333		0,10088497	
I100,Hasanbey	9,820000		0,10088497	
I100,Seçkin	9,660000		0,10088497	
I100,Ýnci	11,153333		0,10088497	
I50,Hasanbey	8,663333		0,10088497	
I50,Seçkin	7,793333		0,10088497	
I50,Ýnci	9,136667		0,10088497	
LSMeans Differences Student's t
Alpha=
0,050 t=
2,17881LSMean[i] By LSMean[j]
Mean[i]-Mean[j]
Std Err Dif
Lower CL Dif
Upper CL Dif	I0,Hasanbey	I0,Seçkin	I0,Ýnci	I100,Hasanbey	I100,Seçkin	I100,Ýnci	I50,Hasanbey	I50,Seçkin	I50,Ýnci	
I0,Hasanbey	0
0
0
0	0,43667
0,14267
0,12581
0,74752	-0,6333
0,14267
-0,9442
-0,3225	-2,94
0,14267
-3,2509
-2,6291	-2,78
0,14267
-3,0909
-2,4691	-4,2733
0,14267
-4,5842
-3,9625	-1,7833
0,14267
-2,0942
-1,4725	-0,9133
0,14267
-1,2242
-0,6025	-2,2567
0,14267
-2,5675
-1,9458	
I0,Seçkin	-0,4367
0,14267
-0,7475
-0,1258	0
0
0
0	-1,07
0,14267
-1,3809
-0,7591	-3,3767
0,14267
-3,6875
-3,0658	-3,2167
0,14267
-3,5275
-2,9058	-4,71
0,14267
-5,0209
-4,3991	-2,22
0,14267
-2,5309
-1,9091	-1,35
0,14267
-1,6609
-1,0391	-2,6933
0,14267
-3,0042
-2,3825	
I0,Ýnci	0,63333
0,14267
0,32248
0,94419	1,07
0,14267
0,75914
1,38086	0
0
0
0	-2,3067
0,14267
-2,6175
-1,9958	-2,1467
0,14267
-2,4575
-1,8358	-3,64
0,14267
-3,9509
-3,3291	-1,15
0,14267
-1,4609
-0,8391	-0,28
0,14267
-0,5909
0,03086	-1,6233
0,14267
-1,9342
-1,3125	
I100,Hasanbey	2,94
0,14267
2,62914
3,25086	3,37667
0,14267
3,06581
3,68752	2,30667
0,14267
1,99581
2,61752	0
0
0
0	0,16
0,14267
-0,1509
0,47086	-1,3333
0,14267
-1,6442
-1,0225	1,15667
0,14267
0,84581
1,46752	2,02667
0,14267
1,71581
2,33752	0,68333
0,14267
0,37248
0,99419	
I100,Seçkin	2,78
0,14267
2,46914
3,09086	3,21667
0,14267
2,90581
3,52752	2,14667
0,14267
1,83581
2,45752	-0,16
0,14267
-0,4709
0,15086	0
0
0
0	-1,4933
0,14267
-1,8042
-1,1825	0,99667
0,14267
0,68581
1,30752	1,86667
0,14267
1,55581
2,17752	0,52333
0,14267
0,21248
0,83419	
I100,Ýnci	4,27333
0,14267
3,96248
4,58419	4,71
0,14267
4,39914
5,02086	3,64
0,14267
3,32914
3,95086	1,33333
0,14267
1,02248
1,64419	1,49333
0,14267
1,18248
1,80419	0
0
0
0	2,49
0,14267
2,17914
2,80086	3,36
0,14267
3,04914
3,67086	2,01667
0,14267
1,70581
2,32752	
I50,Hasanbey	1,78333
0,14267
1,47248
2,09419	2,22
0,14267
1,90914
2,53086	1,15
0,14267
0,83914
1,46086	-1,1567
0,14267
-1,4675
-0,8458	-0,9967
0,14267
-1,3075
-0,6858	-2,49
0,14267
-2,8009
-2,1791	0
0
0
0	0,87
0,14267
0,55914
1,18086	-0,4733
0,14267
-0,7842
-0,1625	
I50,Seçkin	0,91333
0,14267
0,60248
1,22419	1,35
0,14267
1,03914
1,66086	0,28
0,14267
-0,0309
0,59086	-2,0267
0,14267
-2,3375
-1,7158	-1,8667
0,14267
-2,1775
-1,5558	-3,36
0,14267
-3,6709
-3,0491	-0,87
0,14267
-1,1809
-0,5591	0
0
0
0	-1,3433
0,14267
-1,6542
-1,0325	
I50,Ýnci	2,25667
0,14267
1,94581
2,56752	2,69333
0,14267
2,38248
3,00419	1,62333
0,14267
1,31248
1,93419	-0,6833
0,14267
-0,9942
-0,3725	-0,5233
0,14267
-0,8342
-0,2125	-2,0167
0,14267
-2,3275
-1,7058	0,47333
0,14267
0,16248
0,78419	1,34333
0,14267
1,03248
1,65419	0
0
0
0	


Level								Least Sq Mean	
I100,Ýnci	A	 	 	 	 	 	 	11,153333	
I100,Hasanbey	 	B	 	 	 	 	 	9,820000	
I100,Seçkin	 	B	 	 	 	 	 	9,660000	
I50,Ýnci	 	 	C	 	 	 	 	9,136667	
I50,Hasanbey	 	 	 	D	 	 	 	8,663333	
I50,Seçkin	 	 	 	 	E	 	 	7,793333	
I0,Ýnci	 	 	 	 	E	 	 	7,513333	
I0,Hasanbey	 	 	 	 	 	F	 	6,880000	
I0,Seçkin	 	 	 	 	 	 	G	6,443333	

Levels not connected by same letter are significantly different
Response bit kök dað
Whole Model
Summary of Fit
 	 	
RSquare	0,996472	
RSquare Adj	0,992357	
Root Mean Square Error	0,132462	
Mean of Response	5,663333	
Observations (or Sum Wgts)	27	
Tests wrt Random Effects
Source	SS	MS Num	DF Num	F Ratio	Prob > F	
tek	0,07209	0,03604	2	2,0243	0,2470	
eþit	1,96682	0,98341	2	55,2306	0,0012	
eþit*tek&Random	0,07122	0,01781	4	1,0148	0,4381	
sul konu	56,011	28,0055	2	1596,091	<.0001	
sul konu*eþit	1,35756	0,33939	4	19,3425	<.0001	
Residual by Predicted Plot


tek
Leverage Plot

Effect Test
Sum of Squares	F Ratio	DF	Prob > F	
0,07208889	2,0243	2	0,2470	
Denominator MS Synthesis: 
 eþit*tek&Random
Least Squares Means Table
Level	Least Sq Mean	 	Std Error	Mean	
1	5,6077778		0,04447915	5,60778	
2	5,6500000		0,04447915	5,65000	
3	5,7322222		0,04447915	5,73222	

eþit
Leverage Plot

Effect Test
Sum of Squares	F Ratio	DF	Prob > F	
1,9668222	55,2306	2	0,0012	
Denominator MS Synthesis: 
 eþit*tek&Random
Least Squares Means Table
Level	Least Sq Mean	 	Std Error	Mean	
Hasanbey	5,6644444		0,04447915	5,66444	
Seçkin	5,3322222		0,04447915	5,33222	
Ýnci	5,9933333		0,04447915	5,99333	

eþit*tek&Random
Leverage Plot

Effect Test
Sum of Squares	F Ratio	DF	Prob > F	
0,07122222	1,0148	4	0,4381	
Denominator MS Synthesis: 
 Residual
Least Squares Means Table
Level	Least Sq Mean	 	Std Error	
Hasanbey,1	5,6466667		0,07647722	
Hasanbey,2	5,6633333		0,07647722	
Hasanbey,3	5,6833333		0,07647722	
Seçkin,1	5,2733333		0,07647722	
Seçkin,2	5,3700000		0,07647722	
Seçkin,3	5,3533333		0,07647722	
Ýnci,1	5,9033333		0,07647722	
Ýnci,2	5,9166667		0,07647722	
Ýnci,3	6,1600000		0,07647722	

sul konu
Leverage Plot

Effect Test
Sum of Squares	F Ratio	DF	Prob > F	
56,010956	1596,091	2	<.0001	
Denominator MS Synthesis: 
 Residual
Least Squares Means Table
Level	Least Sq Mean	 	Std Error	Mean	
I0	4,2177778		0,04415414	4,21778	
I100	7,6288889		0,04415414	7,62889	
I50	5,1433333		0,04415414	5,14333	

sul konu*eþit
Leverage Plot

Effect Test
Sum of Squares	F Ratio	DF	Prob > F	
1,3575556	19,3425	4	<.0001	
Denominator MS Synthesis: 
 Residual
Least Squares Means Table
Level	Least Sq Mean	 	Std Error	
I0,Hasanbey	4,1200000		0,07647722	
I0,Seçkin	4,0866667		0,07647722	
I0,Ýnci	4,4466667		0,07647722	
I100,Hasanbey	7,7533333		0,07647722	
I100,Seçkin	6,8666667		0,07647722	
I100,Ýnci	8,2666667		0,07647722	
I50,Hasanbey	5,1200000		0,07647722	
I50,Seçkin	5,0433333		0,07647722	
I50,Ýnci	5,2666667		0,07647722	
