# Supplementary material for: Drought stress resistance indicators of chickpea varieties grown under deficit irrigation conditions
Source: PeerJ. 2023 Mar 10;11:e14818. doi: 10.7717/peerj.14818 (PMC10010177; doi:10.7717/peerj.14818)
Supplement: Supplemental Information 5 — Since the experimental design was split plots in randomized blocks, firstly, raw data were entered in Excel in accordance with this experimental design. In other words, the raw data were arranged in such a way that analysis of variance was carried out. In the research, 3 irrigation subjects and 3 genotypes constitute the plant material. Therefore, the data were subjected to analysis of variance in the jump 5.01 computer package program in accordance with the experimental design of divided parcels in random blocks. After the analysis, the value of Probe >F (significance level) was checked in the Test wrt Random Effects table (Below). [file peerj-11-14818-s005.docx]

**Statistical Report**

**Statistical results**

1. **Performed test: Variance analysis was performed in the Jump 5.01 statistical package program.**
2. **Related test statistic: As a result of analysis of variance (p: significance level was checked). Is the materiality level less than 1% or less than 5%? In the important application, if the p value is less than 1%, a double star (**) is put, and if the p value is less than 5%, a single star (*) is put. Following this, the important application was subjected to the LSD (least squar different) test.**

**Since the experimental design was split plots in randomized blocks, firstly, raw data were entered in Excel in accordance with this experimental design. In other words, the raw data were arranged in such a way that analysis of variance was carried out. In the research, 3 irrigation subjects and 3 genotypes constitute the plant material. Therefore, the data were subjected to analysis of variance in the jump 5.01 computer package program in accordance with the experimental design of divided parcels in random blocks. After the analysis, the value of Probe>F (significance level) was checked in the Test wrt Random Effects table (Below).**

**For example) Interpretation of variant in Tests wrt Random Effects table; The prop>F value for the variety was found to be <.0001 and it was found to be significant at the 1% level, and ** was placed next to it. Others have been interpreted similarly.**

**Tests wrt Random Effects**

| Source | SS | MS Num | DF Num | F Ratio | Prob > F |
| --- | --- | --- | --- | --- | --- |
| recurrences | 395,63 | 197,815 | 2 | 0,9840 | 0,4492 |
| vaieties | 229716 | 114858 | 2 | 571,3266 | <.0001 |
| vaieties*recurresRandom | 804,148 | 201,037 | 4 | 0,8748 | 0,5071 |
| İrrigation | 1376265 | 688132 | 2 | 2994,532 | <.0001 |
| irrigation*vaieties | 13397,9 | 3349,48 | 4 | 14,5759 | 0,0001 |

**The accuracy of the analysis of variance was tested by calculating the mean of squares and it was found to be correct. as follows**

**Mean of squares = sum of squares (395,63)/ Degree of freedom (DF) 2 = 197,815 and it was understood that the analysis was correct.**

**Error was obtained by multiplying the repetition and the subject, then the general error was found by adding them all together and it was determined that the operation was correct.**

**Then the coefficient of variation (CV(%)) and lsd (Least significant difference) values were calculated.**

**It is found by dividing the root mean square error/mean of response from the Summary of Fit table.**

**Cv= 15.15903/2171.148 x100 = 0.69820344 about= 0.70**

**For example, finding LSD (0.05) for yield; LSMeans Diffrences Student's (t=2.77645) x 6.68393=15.57 was found.**
